# Quantitative assessment of the impact of partially protective anti-schistosomiasis vaccines

**DOI:** 10.1371/journal.pntd.0005544

**Published:** 2017-04-14

**Authors:** Ramzi A. Alsallaq, David Gurarie, Martial Ndeffo Mbah, Alison Galvani, Charles King

**Affiliations:** 1 Center for Global Health & Diseases, College of Medicine, Case Western Reserve University, Cleveland, Ohio, United States of America; 2 The Department of Mathematics, Applied Mathematics and Statistics, Case Western Reserve University, Cleveland, Ohio, United States of America; 3 Yale School of Public Health, Yale University, New Haven, Connecticut, United States of America; Australian National University, AUSTRALIA

## Abstract

**Background:**

Mass drug administration (MDA) of praziquantel has been the intervention of choice against schistosomiasis but with limited success in interrupting the transmission. The development of anti-*Schistosoma* vaccines is underway. Our objective is to quantify the population-level impact of anti-*Schistosoma* vaccines when administered alone and in combination with mass drug administration (MDA) and determine factors in vaccine design and public health implementation that optimize vaccination role in schistosomiasis control and elimination.

**Methods and findings:**

We developed a deterministic compartmental model simulation of schistosomiasis transmission in a high-risk Kenyan community, including stratification by age, parasite burden, and vaccination status. The modeled schistosomiasis vaccines differed in terms of vaccine duration of protection (durability) and three biological efficacies. These are vaccine susceptibility effect (SE) of reducing person’s susceptibility to *Schistosoma* acquisition, vaccine mortality effect (ME) of reducing established worm burden and vaccine fecundity effect (FE) of reducing egg release by mature worms. We quantified the population-level impact of vaccination over two decades under diverse vaccination schemes (childhood vs. mass campaigns), with different age-targeting scenarios, different risk settings, and with combined intervention with MDA. We also assessed the sensitivity of our predictions to uncertainties in model parameters. Over two decades, our base case vaccine with 80% SE, FE, and ME efficacies, 10 years’ durability, provided by mass vaccination every 10 years, reduced host prevalence, mean intensity, incidence, and patent snail prevalence to 31%, 20 eggs/10-ml sample/person, 0.87 worm/person-year, and 0.74%, from endemic-state values of 71%, 152, 3.3, and 0.98%, respectively. Lower impact was found when coverage did not encompass all potential contaminators, and childhood-only vaccination schemes showed delayed and lower impact. In lower prevalence settings, the base case vaccine generated a proportionately smaller impact. A substantially larger vaccine program effect was generated when MDA + mass vaccination was provided every 5 years, which could be achieved by an MDA-only program only if drug was offered annually. Vaccine impact on schistosomiasis transmission was sensitive to a number of parameters including vaccine efficacies, human contact rates with water, human density, patent snails’ rate of patency and lifespan, and force of infection to snails.

**Conclusions:**

To be successful a vaccine-based control strategy will need a moderately to highly effective formulation combined with early vaccination of potential contaminators and aggressive coverage in repeated rounds of mass vaccination. Compared to MDA-only program, vaccination combined with MDA accelerates and prolongs the impact by reducing the acquisition of new worms and reducing egg release from residual worms.

## Author summary

A number of experimental anti-schistosomiasis vaccines are under development. Our results have implications on the vaccines’ design and implementation. Design: For optimal impact at the population-level we found that the best vaccines are the ones that substantially reduce the acquisition of new worms and that kill existing worms with high potency. The next best vaccines for optimal impact at the population-level were those that both markedly reduced acquisition of new worms and reduced the shedding of eggs from residual worms. Implementation: For the best impact on transmission, we found that mass coverage in repeated rounds is required at an interval less than the mean durability of the vaccine-induced immunity. If the vaccine does not effectively kill existing worms, combining vaccine with MDA in these implementation rounds would compensate. Without vaccination, MDA programs have to be offered at five times higher frequency and for longer periods in order to get comparable impact at the population level.

## Introduction

Schistosomiasis, also known as bilharziasis, is a chronic parasitic disease that is common in the developing world [1]. Although the disease is treatable with the use of praziquantel (PZQ) drug therapy, reinfection following treatment is the norm in the 51 countries where the disease is transmitted [1]. *Schistosoma* infections pose a major global health challenge, with an estimated quarter billion persons in need of treatment as of 2014 [1]. The reality of frequent reinfection after treatment augments the programmatic challenge to affected nations. Thus it will be important to find better means to limit *Schistosoma* transmission in order to drastically reduce the global disease burden of schistosomiasis.

Mass drug administration (MDA) using PZQ is being successfully implemented in many endemic countries. Following WHO recommendations [2] the primary targets for treatment have been school-aged children. At the same time, development of anti-*Schistosoma* vaccines has gained momentum given vaccine’s potential for greater efficacy and longer lasting protection than drug given alone. The basic vaccine strategy focuses on the biological efficacy of the vaccine of protecting vaccinated individuals against acquiring new worms and reducing the risk of primary infection among vaccinated individuals who have never been exposed (i.e., a reduced susceptibility effect, SE). Further, the vaccine could also kill worm parasites (a *therapeutic* vaccine with mortality effect quantified as increased parasite mortality, ME) or reduce egg release from mature female worms (an *anti-fecundity* effect, FE) with concomitant reduction in egg-related morbidities. Thus, effective vaccines that have multiple efficacies over a long duration could have an edge over current MDA approaches to control. When MDA and vaccines are combined, they could provide an even larger impact at the population-level than when used alone.

At least three anti-schistosome vaccines are currently in the pipeline: Sm14 [3], Bilhvax [4], and Sm-TSP-2 [5]. These have undergone Phase 1 testing for immunogenicity and initial safety in volunteers, and have shown robust immune responses with no significant adverse effects. An Sm-TSP-2/Alhydrogel combination is currently being tested by the Sabin Vaccine Institute [6]. The Bilhvax vaccine has been tested in Phase 3 trial in West Africa, and the results are being analyzed. Plans are underway to test the other vaccines in Phase 2 and 3 trials in schistosomiasis-endemic areas in South America and Africa. The three types of biological vaccine efficacy, SE, FE, and ME, and the mean duration of their effects (*D*) are among the expected outcomes to be measured in future Phase 3 vaccine trials of these vaccines.

In anticipation of these forthcoming trials and eventual licensure of a vaccine, we set out to quantify, using mathematical modeling, the potential population-level impact of currently targeted vaccines when used alone or in combination with MDA. Our goal is to inform current vaccine design on the parameters that would maximize their epidemiological impact in future public health implementation. To this end, we have simulated these effects using a model of the transmission-contamination cycle of *Schistosoma haematobium* infection in a high risk community in coastal Kenya, an endemic region in sub-Saharan Africa.

## Methods

### Model structure and parameterization

We constructed a deterministic compartmental model in Python (Enthought Incorporation, Austin, TX) to mathematically describe the transmission-contamination cycle of *Schistosoma haematobium* with and without vaccination in different populations. We based our analyses on a high-prevalence endemic setting in Kenya because model parameters (without vaccination) had been previously estimated for a range of endemic villages in coastal Kenya [7]. The Supporting Information Appendix (S1 Text) provides the details of the model and its parameterization.

In the model (see S1 Fig for cartoon depiction), the population was stratified into compartments according to vaccination status (vaccinated or unvaccinated), age (children 0–4 years old, school-aged children 5–14 years old, young adults 15–24 years old, and older adults 25+ years old), and worm burden with dynamical distribution over strata following a stratified worm burden (SWB) approach (see [7–9]).

The basic theory of stratified worm burden modeling goes back to the work of Kostizin [10] who used an infinite system of coupled differential equations. However, we have used a truncated version and can explicitly represent the process of worm accumulation and the instantaneous variability of worm distributions [7–9].

In the SWB framework, human hosts are divided into worm burden strata {*h*_*k*_: *k* = 0,1,…} defined by increments of worm step Δ*ω* with population in *h*_*k*_(*t*) carrying adult worms in the range *k*Δ*ω* to (*k* + 1)Δ*ω* at time *t*. Transitions between adjacent strata represent the processes of worm accumulation and death. We ignored the short time interval between worm acquisition and the later adult stage when worms are able to mate. The mating worms produce eggs that are shed into the environment by human hosts, which contaminate nearby waters by infecting fresh water intermediate host snails. The rate of worm accumulation and worm fecundity (number of eggs per mated worm per 10ml sample) were assumed to depend on age of host. Worm fecundity was also assumed to decrease with increasing parasite burden (see the table S2 Text).

Demographic data on age-specific mortalities were from Kenya’s demographic and health surveys (KDHS) [11–13], the baseline *in vivo* mortality rate of worm parasites was 0.2 years (reciprocal of 5 years lifespan [14]) and the maximum fecundities of worms (ignoring crowding effect for parasites) were assumed to decrease with host age and were 45, 32, and 11 for years of human host age: <14, between 15 and 24, and ≥25; respectively. For all human host ages, the maximum fecundity was assumed to drop exponentially with the number of parasitic worms due to a crowding effect with a threshold parasitic worm number of 120 worms [7].

We used a simple Susceptible-Exposed-Infectious (SEI) compartmental model for snails with three compartments representing snail’s status of infection: susceptible, pre-patent, and patent. The fraction of patent snails at endemic levels was set in the baseline scenario to ~1% as reported for coastal Kenya in a study that conducted regular snail sampling [15] by adjusting a parameter controlling the movement from pre-patent to patent stages. With the implicit assumption that patent snails die to be instantly replaced by susceptible snails we fixed the snail density with time.

The expected outcomes from Phase 3 anti-schistosomiasis vaccine trials are summarized in Table 1. These reflect the values of three types of vaccine efficacy and the durability of the vaccine effect represented by these efficacies. Vaccine protection by a partially efficacious vaccine is represented in the model by its efficacy in reducing worm accumulation (reduction in susceptibility, SE), its efficacy in increasing worm mortality (ME), its efficacy in reducing the number of eggs produced by mated worms (reduction in fecundity, FE), and the duration of its action (mean time to waning effect, *D*). Because there are no prior data suggesting otherwise, we assumed vaccine efficacies to be independent of age and worm burden.

**Table 1:** Vaccine biological effects.

Outcome	Measured effect at the individual level
SE (susceptibility effect: efficacy of reducing worm accumulation)	% reduction in worm accumulation
FE (fecundity effect: efficacy of reducing eggs released to environment)	% reduction in worm fecundity
ME (mortality effect: efficacy of increasing worms’ *in vivo* mortality)	% increase in worm mortality
*D* (durability)	The mean duration in years of the vaccine effect represented by the efficacies.

Individual level efficacies of potential schistosomiasis vaccines, as could be measured in Phase 3 vaccine randomized controlled trials.

### Modeled interventions and scenarios

For the base case vaccine (BCV) scenario we modeled a vaccine meeting the proposed target product profile of the Sabin Vaccine Institute’s vaccine program, i.e., a vaccine having 80% efficacies (SE = FE = ME = 80%) and a mean duration (*D*) of 10 years [6]. Because of the lack of empirical estimates of the efficacies SE, FE, and ME we studied in the analyses the effect of varying the values of these efficacies on the impact of vaccination.

In our simulations, starting from time zero, vaccination could be implemented either as a regular implementation for newborns before they have any infection (“childhood campaign”), in which a proportion of newborns (*f*_*v*_) are recruited to the vaccinated class each year, or alternatively in a “mass vaccination campaign” in which a proportion of all unvaccinated people, regardless of infection status, transitions to the vaccinated class though a vaccination event. The vaccinated persons remain in the vaccinated category for a mean duration *D* equal to the vaccine waning duration (durability). That is, vaccinated persons move back to the unvaccinated category and become eligible for vaccination in future vaccination rounds at an exponential rate given by the reciprocal of the waning duration (1/*D*).

In childhood campaigns, a proportion of newborns (*f*_*v*_) become vaccinated and this intervention is maintained over the years beginning the year that the campaign started. In the mass vaccination scheme, vaccination of pre-selected unvaccinated age group(s) (according to a pre-defined coverage targets) can be repeated every few years according to a pre-defined frequency.

To simulate diverse endemic settings, we varied the contact rates with water by varying the values of the underlying transmission rates (*α*) in the Table S2 Text, preserving both the pattern of water contacts across age groups and the relative transmission rate from humans to snails (*β*_0_).

In simulating combined interventions of PZQ and schistosomiasis vaccines, we assumed that both are offered regardless of infection status in mass campaigns and that the efficacy of PZQ is killing 75% of adult worms over 28 days [16]. Because of vaccine–mediated boosting of anti-parasite immunity, the combination of PZQ and vaccine is expected to manifest a substantially larger efficacy of killing worms [16].

In the analyses, we studied the effect of varying the presumed efficacies and durability of the base case vaccine effect, we analyzed outcomes in different endemic settings and in scenarios where different age groups were vaccinated, and we examined the joint impact when vaccination and mass drug administration (MDA) were combined. To estimate the possibility of schistosomiasis elimination in modelled scenarios, we adopted the WHO definition of reaching and maintaining zero incidence [17]. Thus, we reported the incidence in terms of the number of new worms acquired per person per year under various scenarios at the short and long terms.

## Results

### Simulation of the impact of the base case vaccine intervention

We projected the population level impact of vaccination over three decades in a high-prevalence *S*. *haematobium*-endemic community using a vaccine with 80% efficacies (SE = FE = ME = 80%) and mean vaccine duration of 10 years. In terms of vaccine schedule, we compared a continuing early childhood vaccination campaign to a mass campaign approach in which vaccination for all ages is repeated every 10 years. In both schedules we assumed universal coverage.

**Fig 1:**
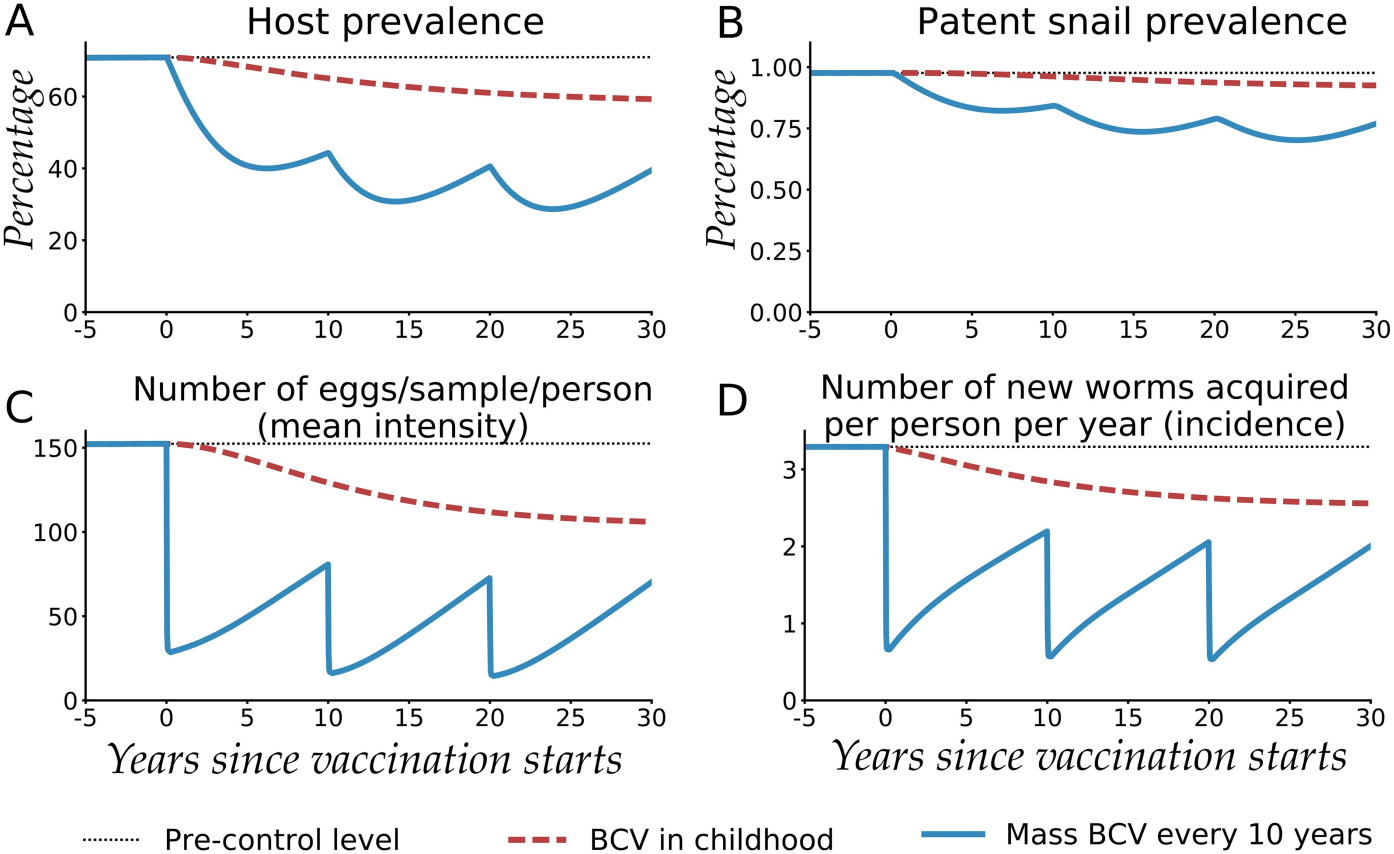
Population-level impact of Base Case Vaccine (BCV) administered in the simulated Kenyan community. The impact is shown on (A) human host prevalence, (B) patent snail prevalence, (C) the number of eggs/10-ml sample/person (mean intensity) and (D) the number of new worms acquired per person per year (incidence). The vaccine’s efficacies are SE = FE = ME = 80%, with a mean duration of protection (*D*) of ten years. Two schedules with universal coverage are shown: mass vaccination every 10 years for three rounds of vaccination (Mass BCV every 10 years) and vaccination of newborns (BCV in childhood). Pre-control endemic values were 71% prevalence, 1% snail patency, 152 eggs/10-ml sample/person mean intensity, and 3.3 worms/person/year incidence.

Following accrual of the long-term effects of vaccination after three rounds of mass vaccination human prevalence (percentage of persons shedding eggs) was reduced by 56%, patent snail prevalence was reduced by 24%, the mean infection intensity was reduced by 87%, and the number of new worms acquired per person per year (incidence) was reduced by 73%, as shown in Fig 1. Because exposure to eggs is the main cause of inflammation and injury, reducing the egg output intensity by 71% should correspond to a substantial reduction in schistosomiasis related morbidity. If we quantify morbidity risk by summing the cumulative ‘egg-years’ experienced by the population, vaccination would prevent a total 2,670 egg-years per person after three rounds of mass vaccination (see S2 Fig).

By contrast, the impact of the same vaccine administered in continuing infant vaccination campaigns developed more slowly and was delayed until the time when a substantial number of vaccinated children had grown up to replace older unvaccinated persons, and contribution of the latter group on transmission dynamics was gradually diminished. These results indicate that by vaccinating only children it would be difficult to effectively interrupt the *Schistosoma* transmission-contamination cycle at an early date. Furthermore, it would be difficult to observe a population-level impact of childhood vaccination in any short-term clinical trials, which typically run for only 2–3 years.

For the mass vaccination scheme, Fig 1 shows that in the interim between vaccine rounds the increasing numbers of incident worms (Fig 1D) as well as increased egg release from incident and existing worms, could lead to gradual reversion to pre-treatment levels of host prevalence and intensity unless vaccination rounds are continued. This is due to the partial efficacy of the vaccine and the ensuing accumulation of persons who lack vaccine protection, either newborn children or those who lose their vaccine-induced immunity.

### Impact at different coverage levels

**Fig 2:**
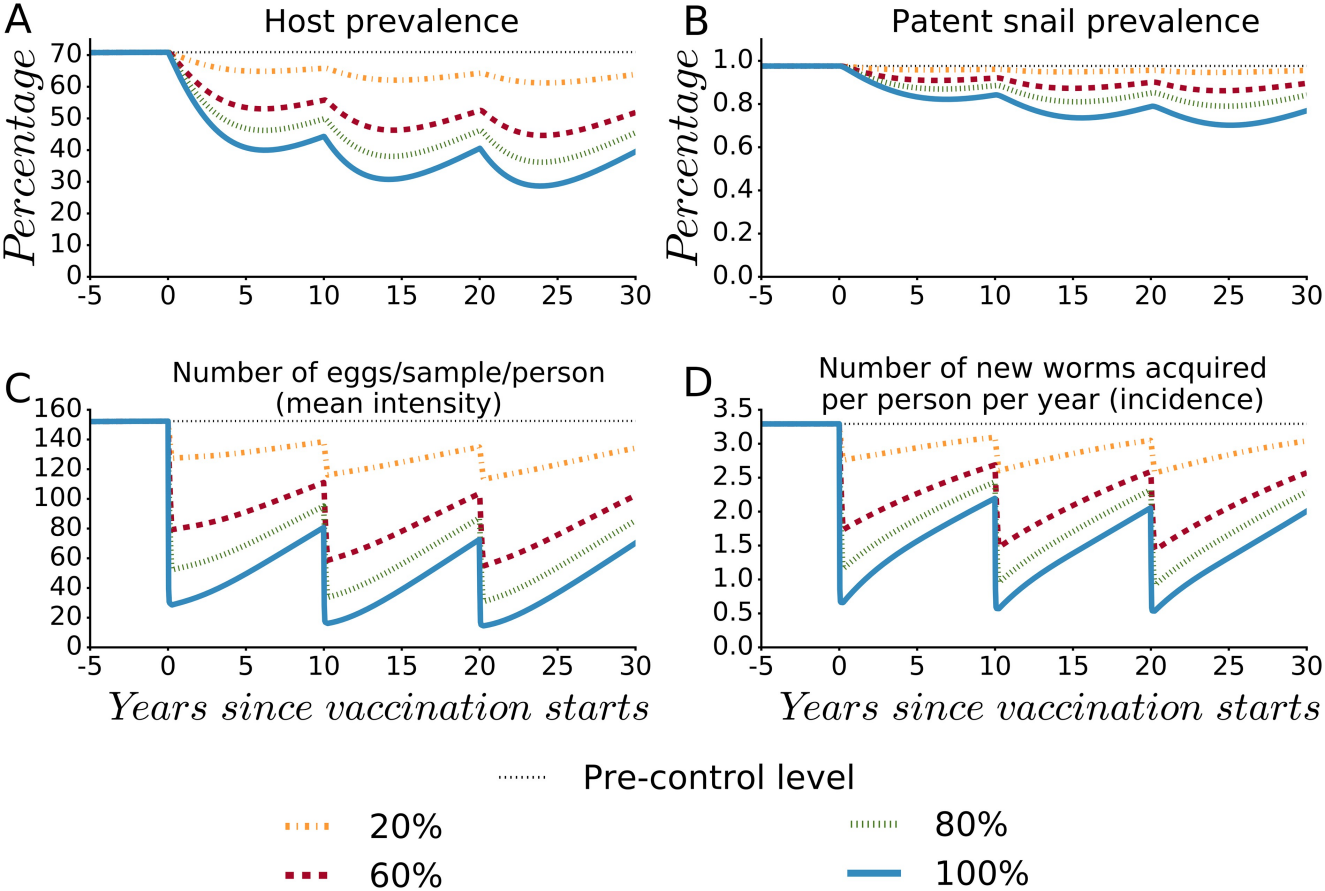
The effect of coverage level attained in mass vaccination rounds on the population-level impact of Base Case Vaccine (BCV). The impact at different coverage levels is shown on (A) human host prevalence, (B) patent snail prevalence, (C) the number of eggs/10-ml sample/person (mean intensity) and (D) the number of new worms acquired per person per year (incidence). The vaccine’s efficacies are SE = FE = ME = 80%, with a mean duration of protection (*D*) of ten years. Rounds of mass vaccination campaigns are assumed every 10 years at coverage levels of 20%, 60%, 80% and universal coverage. The percentage vaccinated is assumed to be randomly assigned.

The impact of the mass vaccination decreases with decreasing coverage in the vaccination rounds as indicated in Fig 2. When vaccination coverage does not include all persons who could circulate the infection, the program impact decreases because transmission to, and contamination from the unvaccinated continues.

### Impact at different administration frequencies and different durability of vaccine effect

**Fig 3:**
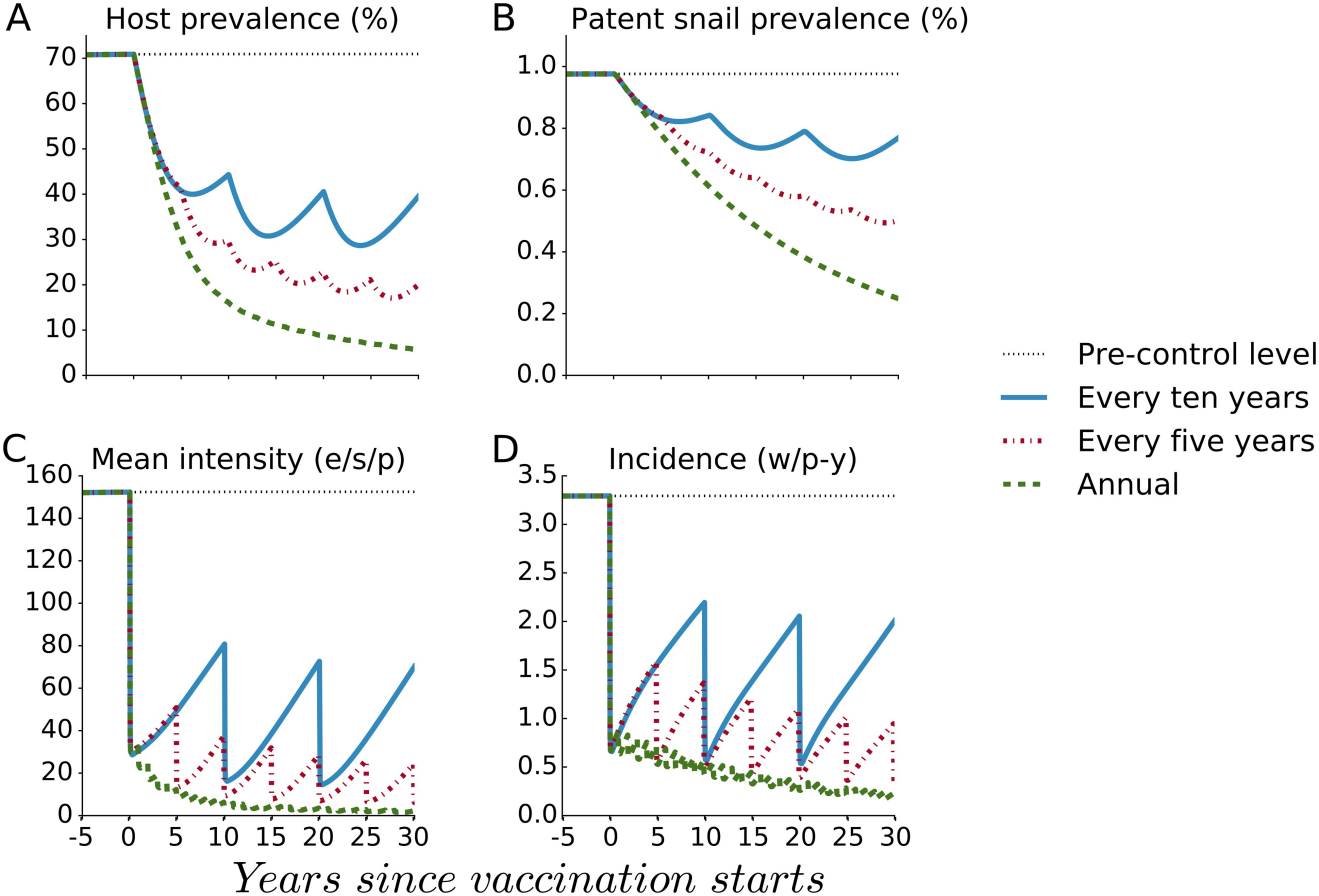
The effect of higher vaccination frequency using the Base Case Vaccine (BCV) on four outcomes. The base case vaccine with a mean duration of protection of ten years (*D* = 10) is given according to different mass vaccination frequencies every ten, five and one years with universal coverage in each round. The panels indicate the impact of vaccination on (A) human host prevalence, (B) patent snail prevalence, (C) mean intensity of human infection (eggs/10-ml sample/person or e/s/p) and (D) incidence measured as the number of new worms acquired per person-year (w/p-y).

**Fig 4:**
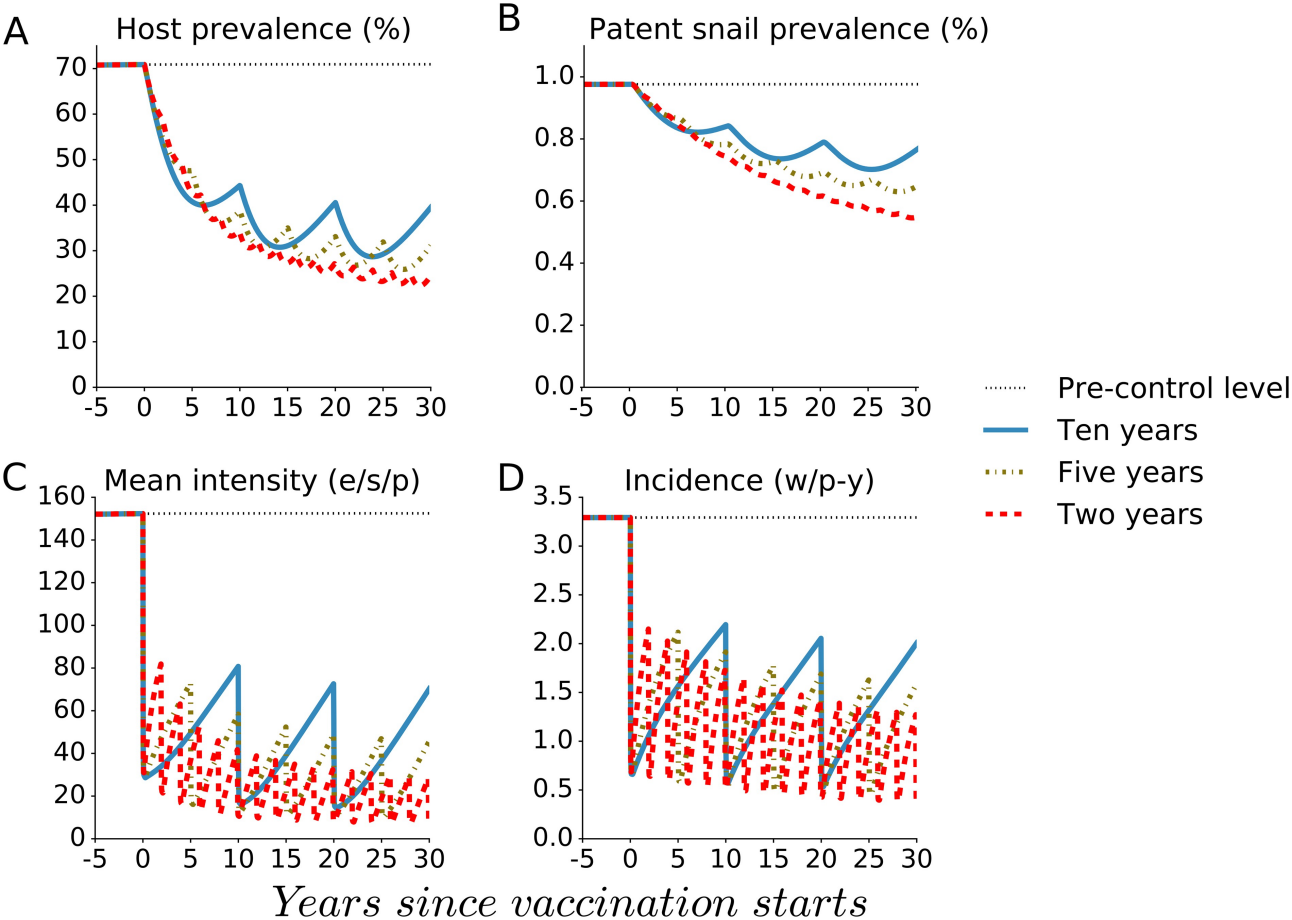
The effect of shorter duration of vaccine effect on four outcomes. The vaccine is assumed to have different durations of effect, either 10, 5, or 2 years, with the mass vaccination administered at frequency intervals set equal to vaccine durability (adaptive vaccination frequency) with universal coverage in each round. The panels indicate the impact of vaccination on (A) human host prevalence, (B) patent snail prevalence, (C) mean intensity of human infection (eggs/10-ml sample/person or e/s/p) and (D) incidence measured as the number of new worms acquired per person-year (w/p-y).

The waning of vaccine impact over time, as indicated in Fig 1, suggests that mass vaccination should be administered at higher frequency for larger impact. Further, if the vaccine had a shorter durability than the assumed 10 years, mass vaccination would have to be administered at shorter intervals to obtain the same impact on infection outcomes. Fig 3 indicates that a more frequent provision of the base case vaccine with 10-year durability in mass vaccination with universal coverage in each round would result in greater impact on human and snail prevalence, on mean infection intensity and on incidence over time. Particularly, annual universal vaccination using BCV drives incidence decline towards elimination. Fig 4 indicates that a vaccine with shorter duration of protection would also have substantial impact as long as the frequency of universal mass vaccination were adapted to the shorter vaccine waning period. In part, this is because increasing the frequency of mass vaccination decreases the proportion of the population who lack any vaccine protection, and it shortens the interval during which they lack this protection.

### Impact of targeting specific age groups for vaccination

**Fig 5:**
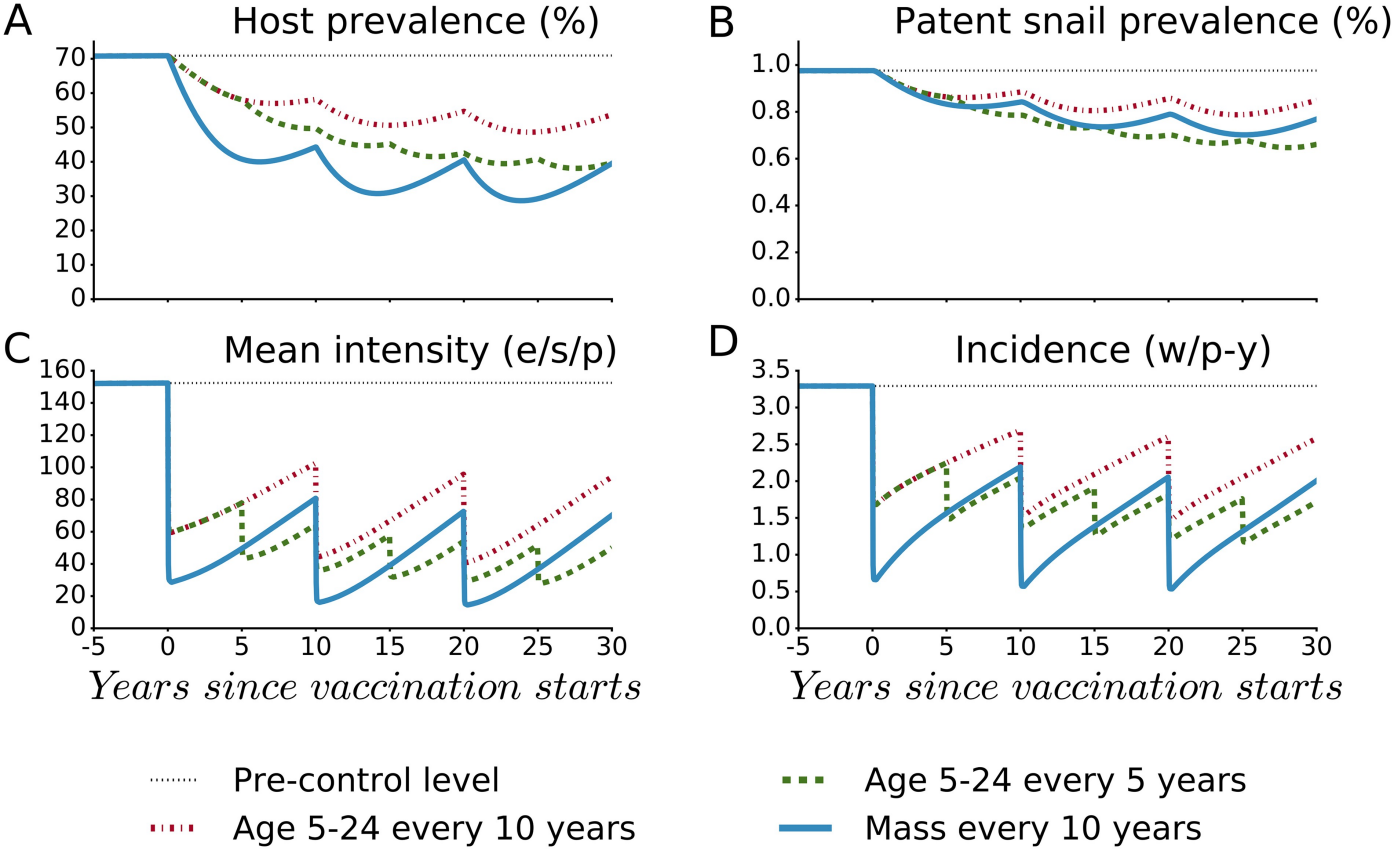
The effect of sub-maximal coverage when vaccination is targeted to high-risk age groups. Our predictions comparing a mass vaccination schedule with universal coverage (blue solid line, corresponding to predictions in Fig 1) versus targeted universal vaccination of persons of age 5–24 every 10 years (red dash-dotted line) or every 5 years (green dotted line). The panels indicate the impact of vaccination on (A) human host prevalence, (B) patent snail prevalence, (C) mean intensity of human infection (eggs/10-ml sample/person or e/s/p) and (D) incidence measured as the number of new worms acquired per person-year (w/p-y).

The greater role of school-aged children and young adults in parasite transmission (represented in our simulations by the age-related variation of the underlying transmission rates (*α*) in the Table S2 Text) suggests that the same impact might be obtainable by targeting vaccination to those age groups who are at highest risk. To investigate this, we varied the vaccination schedule in the base case scenario (mass vaccination every 10 years with universal coverage) to universally vaccinate school-aged children and young adults (all persons 5–24 years of age) either every 5 or every 10 years. Fig 5 indicates that the population-level impact of vaccination campaigns on mean intensity is slightly larger on average when targeting 5–24 year-olds with frequent vaccination every 5 years as reflected in patent snail prevalence. However, the impact on prevalence, and incidence is decreased substantially when coverage is targeted to smaller groups, even with more frequent vaccination. When vaccination coverage does not include all persons who circulate the infection, the program impact decreases because transmission to, and contamination from, younger and older unvaccinated age groups are not directly affected.

### Impact of vaccination in diverse endemic settings

We next examined impact of the base case vaccine in different endemic settings if mass vaccination were to be administered every ten years with universal coverage. These different endemic settings were characterized by different human water contact rates (see Methods), in which communities with higher water contact rates had higher pre-vaccination infection intensity and prevalence among humans, and higher pre-vaccination patent snail prevalence.

**Fig 6:**
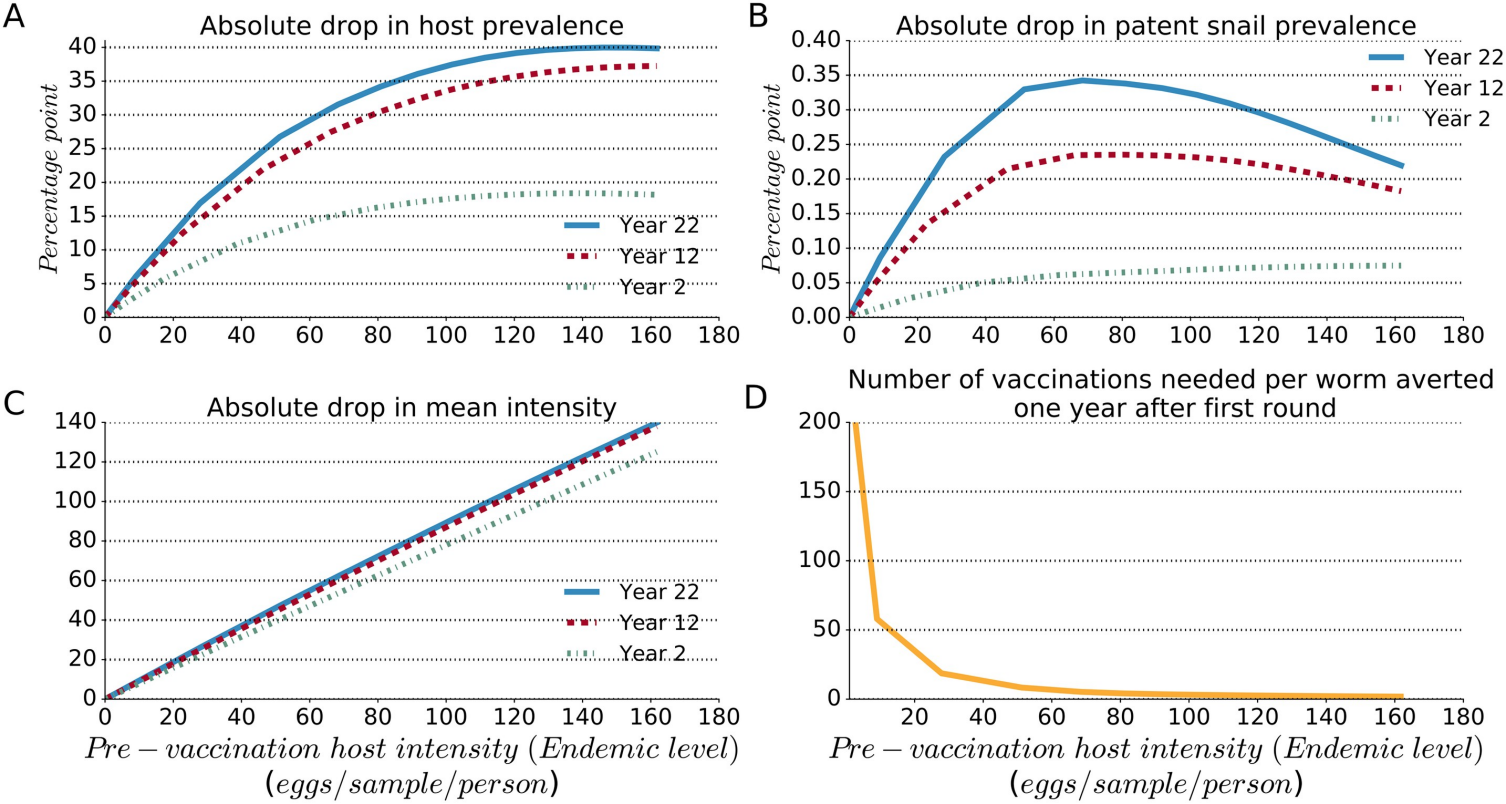
Predicted impact of mass vaccination with universal coverage in diverse schistosomiasis endemic settings at years 2, 12 and 22 following one, two, or three mass vaccination rounds, respectively. By increasing the transmission parameters, different endemic settings with different contact rates—represented by the pre-vaccination host intensity in the abscissa—were generated. The absolute drop in host prevalence (panel A), absolute drop in patent snail prevalence (panel B), and the absolute drop in mean number of eggs/10-ml sample/person (panel C) are calculated at years 2, 12 and 22 by subtracting the value post-intervention from the value pre-intervention. Panel D shows the number of vaccinations needed in the first round to prevent the accrual of one new worm by persons in the community during one year after the first round of vaccination.

As shown in Fig 6, the higher the community transmission risk, the larger the absolute drop in host prevalence and intensity that occurs following vaccination intervention (value pre-vaccination minus value post-vaccination) (Fig 6A and 6C). However, the proportional drop in patent snail prevalence, which grew with increased pre-vaccination intensity up to 50–80 eggs/10-ml sample/person (and a corresponding 34–48% host prevalence), decreased at higher levels of endemicity, especially over time. This indicated a diminishing impact of vaccination on snail prevalence (and consequently a diminishing indirect impact on transmission to humans) over the long term in endemic settings of relatively high host intensity and prevalence. In contrast, the absolute drop in host prevalence and intensity grew consistently larger with continued vaccination in settings with higher contact rates, especially after the second round of mass vaccination (year 12).

The number of persons needed to vaccinate to prevent the accrual of one new worm by persons in the general community (the number of vaccinations needed per worm averted) decreased from 58 to 3 vaccinations as the modeled pre-vaccination endemic intensity was increased from 9.0 to 111.0 eggs/10-ml sample/person (corresponding, respectively, to 7.0% and 60% human host prevalence).

These results indicate that mass vaccination would be more cost-effective in terms of parasite reduction in heavily infected areas. However, the results suggest that over time, even with continued rounds of mass vaccination, there is a diminishing impact on patent snail prevalence in endemic settings with higher pre-vaccination levels of human intensity and prevalence. The implication is that further interventions would be necessary in high intensity settings for substantial long-term impact on patent snail prevalence.

### The effect of varying vaccine efficacy

**Fig 7:**
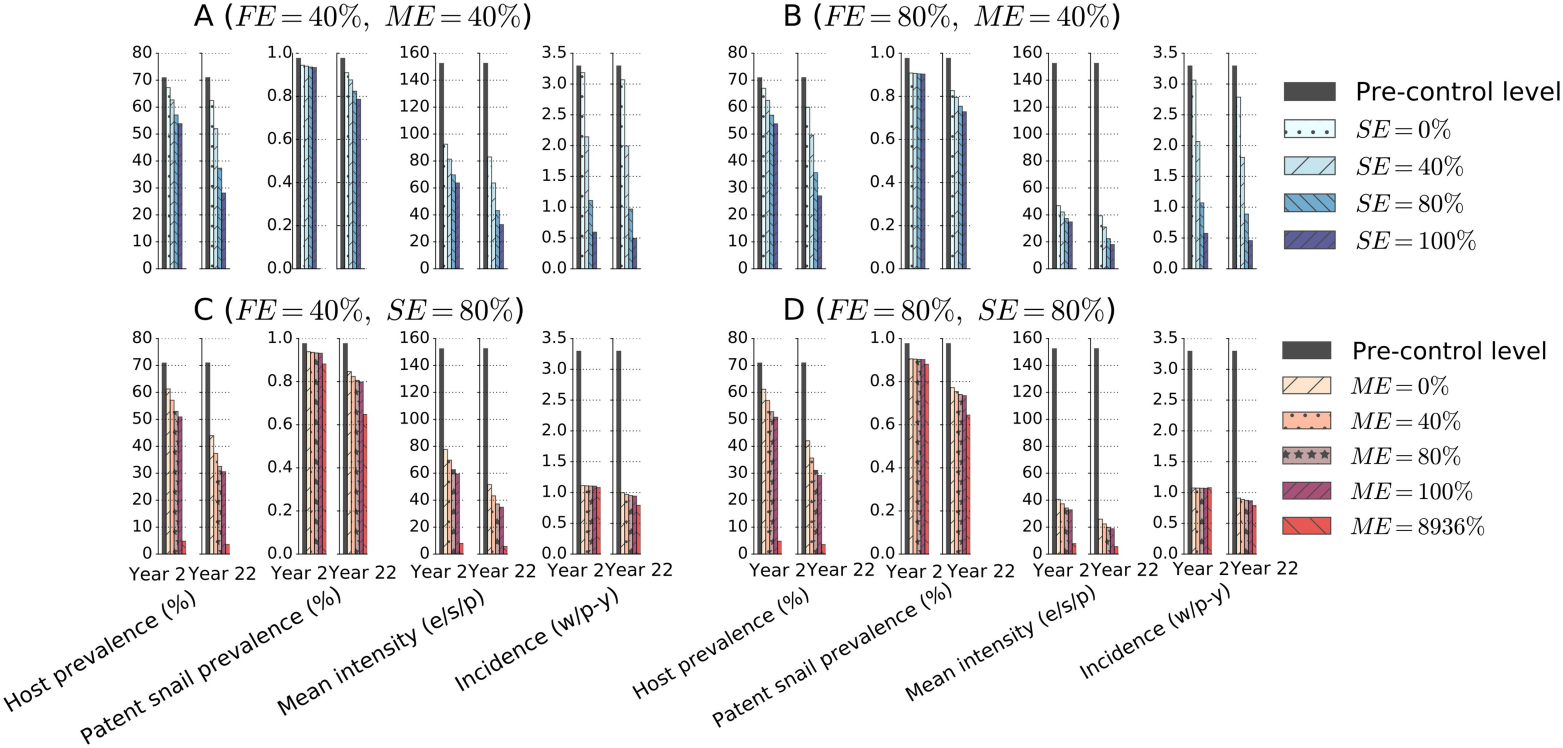
The impact of vaccination in terms of four outcomes in the short-term (year 2) and the long-term (year 22) at different assumptions of vaccine efficacies. The vaccine durability and the frequency of mass vaccination are both assumed to be 10 years assuming universal coverage in each round of vaccination. The unit (e/s/p) stands for the number of eggs per 10ml sample per person and (w/p-y) stands for the number of new worms acquired per person-year.

We next studied the population-level effect of variation in the three potential vaccine efficacies, SE, FE, and ME. In order to understand their relative roles, we studied the short- (at year 2) and long-term (at year 22) population-level impact of varying the vaccine efficacies of the base case vaccine assuming a 10-yearly universal mass vaccination strategy. Increasing the vaccine efficacy of reducing worm accumulation (SE) from 0% to 100% reduction in larval survival (Fig 7A), for a vaccine with limited efficacy in terms of reducing fecundity (FE) and increasing worm mortality (ME), boosted overall vaccine impact in both the short and the long term. The impact of SE on incidence (rate of worm accumulation) is immediate while its impacts on established human prevalence and intensity and on patent snail prevalence accrue over time.

At the desired high efficacy of reducing worm accumulation (SE = 80%), increasing the vaccine efficacy of reducing fecundity (FE) from a minimal efficacy target of 40% to the desired efficacy of 80% (Fig 7B) generates more impact on all outcomes except on established host prevalence, for which less impact is expected than if we increased ME to 80% instead, while keeping FE at 40% (Fig 7C).

When SE and FE are both high (80%), increasing the efficacy of killing worms (ME) from 0% to 100% increases the impact of vaccination on all outcomes provided that there is continued vaccination intervention (Fig 7D). However, if it is possible to increase the vaccine efficacy of killing worms to levels comparable to chemotherapy’s efficacy bringing sharp and immediate reduction in egg-release, then the population-level impact would not substantially change when comparing between scenarios of marginal and high values of FE (comparing between Fig 7C and 7D panels).

These results indicate that the efficacies SE and FE are driving the short-term impact of the vaccine on all outcomes except on host prevalence which is driven by SE and ME. When the vaccine efficacy of killing worms is small compared to chemotherapy’s potency, the long term impact of the vaccine in the modelled community is mostly affected by SE and FE, although their impact increases with increasing ME. A high ME that is comparable to chemotherapy, combined with a high SE would accentuate the impact of vaccine on all outcomes at all times.

### The impact of combined vaccination and Mass Drug Administration (MDA)

To examine the potential impact of combined drug-treatment and vaccination strategies, we next evaluated the following six scenarios of combined intervention (vaccine + PZQ) offered regardless of human infection status assuming universal coverage in intervention rounds. The first three focused on school-aged children consistent with current recommendations for PZQ administration, and the remaining three simulations were for community wide interventions. The first was an every 5-year campaign of PZQ offered to school-aged children in combination with a vaccine having 40% efficacy and 5-year durability. The second was every ten-year campaign of PZQ offered to school-aged children in combination with our base case vaccine having 80% efficacies and 10-year durability. The third was the same intervention but repeated every 5 years. The fourth was an every 10-year mass campaigns of PZQ offered in combination with base case vaccine. The fifth was the same as the fourth but repeated every 5 years, and the sixth was the same as the fourth, but repeated annually.

**Fig 8:**
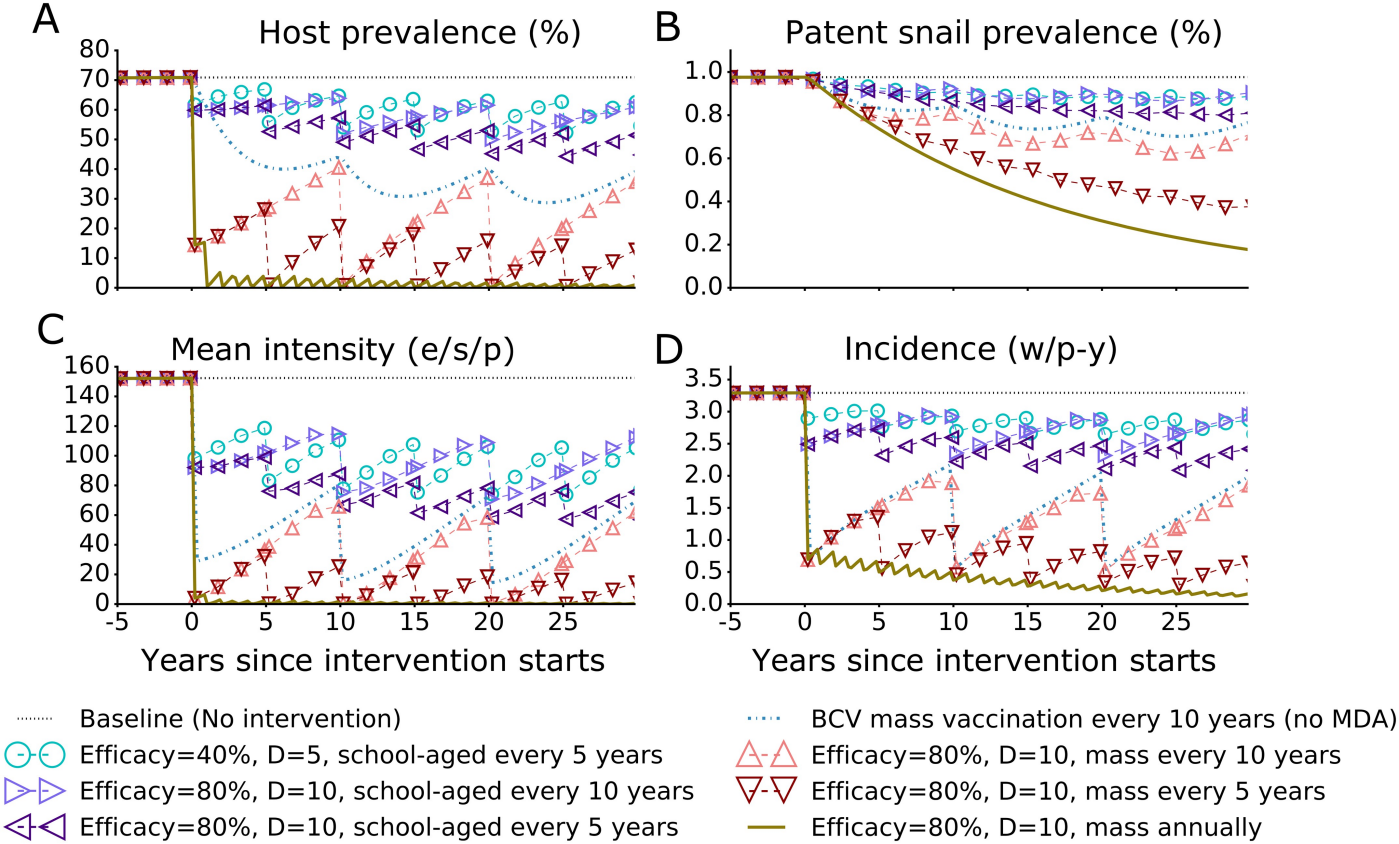
The impact of combined intervention (PZQ + vaccine) on four outcomes. The panels indicate the impact of different interventions on (A) human host prevalence, (B) patent snail prevalence, (C) mean intensity of human infection (eggs/10-ml sample/person or e/s/p) and (D) incidence measured as the number of new worms acquired per person-year (w/p-y). Vaccine durability in years is indicated in the legend by the value of *D*. For comparison we included a scenario of base case vaccine (BCV) without MDA (a vaccine with 80% efficacy and 10-year durability offered in mass campaigns every 10 years) shown as blue dashed line without markers. Universal coverage is assumed in each round of intervention. To compare the additional impact of vaccines relative to PZQ-only interventions, a corresponding figure without vaccine intervention (PZQ-only) is provided in S3 Fig.

As indicated in Fig 8, the joint impact on human host prevalence, mean intensity and incidence and on patent snail’s prevalence increases with the efficacy and the mean duration of the vaccine effect, and with coverage and frequency of the combined intervention of vaccine + PZQ. A mass administration of base case vaccine in combination with PZQ every 10 years, when compared to the impact of offering the vaccine alone, substantially increases program impact in the interim years following each round, particularly for human prevalence (Fig 8 upward triangles compared with dash-dotted line). Increasing the frequency of this combined intervention to every 5 years (Fig 8 downward triangles) could substantially decrease host prevalence, patent snail prevalence, mean intensity and incidence over five rounds of combined intervention to very low levels: 6.13%, 0.44%, 5.2 eggs/10-ml sample/person and 0.54 worms/person-year in year 22; respectively.

To obtain comparable low levels of host prevalence, mean intensity and patent snail’s prevalence in year 22 without vaccinating the simulated community, MDA would have to be provided annually (S4 Fig). Compared to PZQ-only, vaccine + PZQ strategies accelerate the reduction in incidence generating a substantial higher impact that starts to accrue immediately following the first round of combined intervention. The additional impact from the anti-schistosomiasis vaccines when added to PZQ facilitate faster elimination by effectively and immediately lowering incidence and therefore require less administration frequencies to get the same impact when compared to PZQ-only strategies. Thus, a vaccine similar to the base case vaccine, when combined with MDA, could reduce the needed frequency of MDA rounds by one fifth while reducing schistosomiasis incidence and intensity to very low levels. Substantial reductions in host prevalence and mean intensity (below 3% and 1.0 egg/10-ml sample/person) can be reached after two rounds of annual mass intervention using base case vaccine combined with MDA (Fig 8 solid line). However, to substantially reduce patent snail prevalence and drive human incidence to zero, many more rounds of combined mass intervention would be required.

### Sensitivity analyses

We studied the sensitivity of our predictions about vaccine impact by varying the base case scenario in terms of parameter assumptions of human population density (500 to 10000), the worm burden increment (5 to 15), the threshold for density dependent fecundity (2 to 500), the maximum fecundity of worms in older adult hosts (1 to 45). Although the latter is assumed to be small in the default analyses, the relatively large fraction of the population in this age group (a little above 50%) and the many years spent in this age class could affect predictions. We also varied the parameter controlling the rate of patency for exposed snails (8 to 33), the force of infection to snails by 50% to 400% of base case value, and patent snails’ lifespan (the reciprocal of turnover rate) from 2 weeks to 5 months.

Our short and long term predictions under vaccination were robust against the assumed variations in the threshold for density dependent fecundity. The short and long term predictions on human prevalence and mean intensity and on patent snail prevalence under vaccination have relatively small variations (up to 30%) against variations in human density and force of infection to snails, they were largely influenced by variations in the patent snail’s lifespan and in snail’s rate of patency; even more so at the short term. Further, predicted impact on mean intensity under vaccination is affected (even more at the short term) by variations in the maximum fecundity in older adult hosts and in the worm burden increment (S5 Fig).

The short term predictions on human incidence under vaccination are influenced by variations in worm burden increment, and snail’s rate of patency and lifespan whereas long term prediction on human incidence was largely influenced by the assumed variations in human density, force of infection to snails, worm burden increment, and snail’s rate of patency and lifespan.

## Discussion

Although it is possible to measure beneficial individual-level effects in clinical trials using either mass or childhood campaigns, our results indicate that high efficacies at the individual level may not readily translate into population-level impact. Our results indicate that the population-level impact of a candidate anti-*Schistosoma* vaccine in a typical schistosomiasis-endemic Kenyan community increases with increasing vaccination coverage among potential water contaminators, and with the continuation of high coverage over the years. Thus, we found that a sufficiently high coverage is not obtained when administering vaccines by means of age-selective or childhood targeting of immunization campaigns, irrespective of the efficacy of the vaccine. Our results suggest that mass vaccination in high burden settings will be necessary to obtain measurable population-level impact at the short term, and that for substantial impact at the long term repeated rounds of mass vaccination would be required. Also, our results highlight the interplay between vaccination coverage and vaccine durability; the longer the durability of vaccine effect the more feasible it is to maintain the necessary high coverage among potential contaminators by conducting rounds of mass vaccination at intervals less than the vaccine durability to attain the desired reductions in *Schistosoma* infection. These findings have implications for vaccine’s public health implementation.

Our results also have implications for vaccine designs that intend to markedly reduce schistosomiasis transmission. For optimum impact at the population-level we found that the best vaccines are the ones that substantially reduce the acquisition of new worms and kill existing worms with high potency comparable to the potency of chemotherapeutic drugs such as PZQ. By reducing the number of newly acquired and the number of existing worms and concomitantly the number of eggs released to environment, such vaccines would be beneficial for faster interruption of the transmission cycle. The next best vaccines in our analyses were those that both markedly reduced acquisition of new worms and reduced the shedding of eggs from residual worms. To optimize the population-level impact of vaccines that do not effectively kill existing worms, our results suggest combining the vaccines with MDA in periodic mass vaccination rounds to practically have high efficacy of killing worms.

The stratified worm approach allowed us to explicitly represent the processes of worm accumulation and infection of snails which facilitated representing the impact of vaccine efficacies in reducing worm accumulation SE and in reducing fecundity FE in the transmission-contamination cycle.

We have assumed the number of snails to be stable, on average, over time because our study did not aim to account for the effect of seasonality in our simulations of the infection cycle. This is an area worthy of further study, because in seasons of increasing numbers of snails, the force of infection to humans will transiently increase over time, leading later to increases in the rates of snail infection, and a periodic ‘forced’ increase in the transmission process that could augment persistence of endemicity. In principle the seasonality would affect the design of schistosomiasis control programs in certain ways that maximize the role of vaccination in combination with PZQ to effectively control acquired parasites and prevent the acquisition of new ones. The durability of the vaccine is expected to be an important factor to consider. Along these lines, if the vaccine durability is comparable to the duration of high transmission seasons; it would make sense to treat people and vaccinate them shortly before the start of high seasons.

Our model is parametrized to represent S.haematobium transmission and our results might not be directly applicable to S.mansoni transmission due to possible differences in model parameters representing the two infections. Future analysis would investigate the impact of vaccination on S.mansoni transmission and elimination.

Our finding that high vaccination coverage is necessary for substantial impact on the transmission cycle in endemic areas is in line with the findings of a recent study estimating the impact of MDA in similar settings [18]. That study indicated that expanding drug coverage beyond infant and school-aged children will be necessary to reach the elimination of schistosomiasis. However, our model indicates that because of persistent acquisition of new worms, it would be difficult for MDA alone to eliminate *Schistosoma* infections from our modeled community. High MDA coverage with supplemental interventions, such as protective vaccines, will be necessary to interrupt the persistent influx of new worms. Our current results showed that compared to MDA or vaccination alone, combining BCV with MDA substantially reduced the required number of rounds needed to attain targeted levels of reduction in host prevalence and mean intensity. When combined intervention was repeated annually, it was possible to very quickly reach a very low incidence of infection and put the disease on the path approaching elimination. Beside indicating that vaccination can act as ‘elimination-accelerator’, our results indicate that vaccination can act as a ‘drug-extender’ i.e., that mass BCV+MDA treatment can be given as little as every five years in order to maintain levels of human and snail prevalence that would otherwise require MDA at annual frequency of administration.

From the control perspective, the projected results of our simulations suggest a potential role for vaccines that have a longer duration of effect, even if they are only partially protective. When such vaccines are administered in mass campaigns in combination with PZQ, reductions to very low levels of *Schistosoma* prevalence are definitely within reach. However, for high-burden regions our results also highlight the requirement for continuing the combined intervention over the years in order to reach the desired reductions in infection outcomes. The economic considerations for such extended program would need further research.

## Supporting information

S1 Text Details of the mathematical model. (DOCX)

S2 Text Model inputs. (DOCX)

S1 Fig Cartoon depiction of model structure. Connector lines indicate coupling between the unvaccinated (top), vaccinated (center) and snail (bottom) compartments. Vaccination is implemented in childhood campaigns via a fraction *f*_*v*_ and in mass campaigns via a rate that is indicated by the downward thick arrow. Vaccinated individuals can leave their vaccinated class to return to the unvaccinated class as indicated by the upward thick arrow.
(EPS)

S2 Fig Population-level impact of Base Case Vaccine (BCV) on egg-years as a surrogate to morbidity. The vaccine’s efficacies are SE = FE = ME = 80% and it confers a mean duration of protection (*D*) of 10 years. Two schedules are shown: mass vaccination every 10 years for three rounds of vaccination (“Mass BCV every 10 years”) and universal vaccination of newborns (“BCV in childhood”). The assumed size of the population is 1000.
(TIF)

S3 Fig The impact of PZQ offered selectively to school-aged children or as community-wide MDA on four outcomes. The panels indicate the impact of vaccination on (A) human host prevalence, (B) patent snail prevalence, (C) mean intensity of human infection (eggs/10-ml sample/person or e/s/p) and (D) incidence measured as the number of new worms acquired per person-year (w/p-y). For comparison we include a scenario of base case vaccine (BCV, a vaccine with 80% efficacy and 10-year durability offered in mass campaigns every 10 years) without MDA. This is shown by a blue dotted line.
(TIF)

S4 Fig The impact of combined intervention vaccine + PZQ offered as a community-wide intervention every five years compared to community wide annual MDA without vaccine. The panels indicate the impact of different interventions on (A) human host prevalence, (B) patent snail prevalence, (C) mean intensity of human infection (eggs/10-ml sample/person or e/s/p) and (D) incidence measured as the number of new worms acquired per person-year (w/p-y). For comparison we include a scenario of base case vaccine without PZQ as a blue dotted line (BCV, a vaccine with 80% efficacy and 10-year durability offered in mass campaigns every 10 years).
(TIF)

S5 Fig The univariate sensitivity of four model outcomes under vaccination (mass vaccination by BCV every 10 years) in the short-term (years 2) and long-term (year 22) to variations of model assumptions. The panels indicate model prediction sensitivity in (A) human host prevalence, (B) patent snail prevalence, (C) mean intensity of human infection (eggs/10-ml sample/person or e/s/p) and (D) incidence measured as the number of new worms acquired per person-year (w/p-y).
(TIF)
