# Supplementary material for: Quantitative assessment of the impact of partially protective anti-schistosomiasis vaccines
Source: PLoS Negl Trop Dis. 2017 Apr 14;11(4):e0005544. doi: 10.1371/journal.pntd.0005544 (PMC5406007; doi:10.1371/journal.pntd.0005544)
Supplement: S1 Text — (DOCX) [file pntd.0005544.s001.docx]

# Supporting Information Appendix

# Model summary

The model used in this study is a deterministic, compartmental model having age, worm burden, and vaccination-status stratification [1,2]. Each age group ($j$) is stratified by adult parasite worm burden to a number of compartments ($k=0,1,2,....,K$) differing successively by a worm burden increment ($\Delta\omega$). This worm burden stratum increment ($\Delta\omega$) is chosen according to the worm burden number below which human hosts do not shed eggs. Thus, the first worm burden compartment ($k=0$) represents the population from that age with number of worms below mating threshold (no eggs produced from that population). Another interpretation for the quantity ($\Delta\omega$) is the mean number of worms acquired per person per contact with water containing patent (cercaria-shedding) snails. The highest stratum number $K$ is set high enough such that the whole spectrum of human worm burden is represented. Explanation on how the value of$K$is selected is given below.

The youngest age group ($j$=0) has a source term that represents the number of newborns entering that age group. Each subsequent age group has source terms represented by the number of population aging from the preceding age group. The stationary distribution of the population in each age group is obtained by integrating the system of age group equations for a time interval sufficiently long that the number in each age group reaches a stationary value. These values are then used to initialize the demography for simulation runs.

Modeling of vaccination effects is implemented by further stratification of the population to vaccinated and unvaccinated groups. Two parameters ($\eta_{h_{0,j,k}}$and $\zeta_{h_{1,j,k}}$) control, respectively, the fraction of the population that get vaccinated from the stratum $h_{0,j,k}$ and the rate of reversion from the vaccinated category $h_{1,j,k}$ to the unvaccinated category $h_{0,j,k}$ (the rate of vaccine effect loss). Thus, the mean duration of vaccine effect is given by ($1/\zeta_{h_{1,j,k}}$).

# Model Equations

The state variables are represented by the population number $h_{v,\mathrm{j},\mathrm{k}}$ in the stratum $(v,j,k)$. The indices $v=0,1$ represent unvaccinated and vaccinated population; $j$=0,1,2,3 represent the age groups (children 0-4 year-old, school aged 5-14 year-old, young 15-24 year-old, and old 25 and above year-old); and $k=0,1,2,....,K$represent the worm burden strata numbers ($0,\Delta\omega,2\Delta\omega,3\Delta\omega,...,K\Delta\omega$). With vaccination, the community population can be viewed as coupled stratified worm burden (SWB) systems for which each SWB system representing a group of the same age interval and with the same vaccination status. Below, we follow this structuring because it simplifies understanding the model equations and relates them to the previously published SWB formalism [2,3]. The supplementary file S1 Fig depicts model structure.

$SWB_{0}$ and $SWB_{0v}$ (unvaccinated and vaccinated children):

----(1)

$SWB_{1}$ and $SWB_{1v}$ (unvaccinated and vaccinated school-aged kids):

$\begin{matrix} \frac{dh_{v,1,0}(t)}{dt}=\tau_{0}h_{v,0,0}-(\lambda_{h_{v,1,0}}+\mu_{1}+\tau_{1})h_{v,1,0}+\gamma_{h_{v,1,1}}h_{v,1,1}+[(-1)^{v}\zeta_{h_{1,1,0}}h_{1,1,0}+(-1)^{v+1}\eta_{h_{0,1,0}}h_{0,1,0}]_{t\geq T_{v}} \\ \frac{dh_{v,1,k}(t)}{dt}=\tau_{0}h_{v,0,k}+\lambda_{h_{v,1,\mathrm{k}-1}}h_{v,1,\mathrm{k}-1}-(\lambda_{h_{v,1,k}}+\mu_{1}+\tau_{1}+\gamma_{h_{v,1,k}})h_{v,1,k}+\gamma_{h_{v,1,k+1}}h_{v,1,k+1}+[(-1)^{v}\zeta_{h_{1,1,k}}h_{1,1,k}+(-1)^{v+1}\eta_{h_{0,1,k}}h_{0,1,k}]_{t\geq T_{v}} \\ \frac{dh_{v,1,\mathrm{K}}(t)}{dt}=\tau_{0}h_{v,0,\mathrm{K}}+\lambda_{h_{v,1,\mathrm{K}-1}}h_{v,1,\mathrm{K}-1}-(\mu_{1}+\tau_{1}+\gamma_{h_{v,1,\mathrm{K}}})h_{v,1,\mathrm{K}}+[(-1)^{v}\zeta_{h_{1,1,K}}h_{1,1,\mathrm{K}}+(-1)^{v+1}\eta_{h_{0,1,K}}h_{0,1,\mathrm{K}}]_{t\geq T_{v}} \end{matrix}$-----(2)

$SWB_{2}$ and $SWB_{2v}$ (unvaccinated and vaccinated young adults):

$\begin{matrix} \frac{dh_{v,2,0}(t)}{dt}=\tau_{1}h_{v,1,0}-(\lambda_{h_{v,2,0}}+\mu_{2}+\tau_{2})h_{v,2,0}+\gamma_{h_{v,2,1}}h_{v,2,1}+[(-1)^{v}\zeta_{h_{1,2,0}}h_{1,2,0}+(-1)^{v+1}\eta_{h_{0,2,0}}h_{0,2,0}]_{t\geq T_{v}} \\ \frac{dh_{v,2,k}(t)}{dt}=\tau_{1}h_{v,1,k}+\lambda_{h_{v,2,\mathrm{k}-1}}h_{v,2,\mathrm{k}-1}-(\lambda_{h_{v,2,k}}+\mu_{2}+\tau_{2}+\gamma_{h_{v,2,k}})h_{v,2,k}+\gamma_{h_{v,2,k+1}}h_{v,2,k+1}+[(-1)^{v}\zeta_{h_{1,2,k}}h_{1,2,k}+(-1)^{v+1}\eta_{h_{0,2,k}}h_{0,2,k}]_{t\geq T_{v}} \\ \frac{dh_{v,2,\mathrm{K}}(t)}{dt}=\tau_{1}h_{v,1,\mathrm{K}}+\lambda_{h_{v,2,\mathrm{K}-1}}h_{v,2,\mathrm{K}-1}-(\mu_{2}+\tau_{2}+\gamma_{h_{v,2,\mathrm{K}}})h_{v,2,\mathrm{K}}+[(-1)^{v}\zeta_{h_{1,2,K}}h_{1,2,\mathrm{K}}+(-1)^{v+1}\eta_{h_{0,2,K}}h_{0,2,\mathrm{K}}]_{t\geq T_{v}} \end{matrix}$----(3)

$SWB_{3}$ and $SWB_{3v}$ (unvaccinated and vaccinated older adults):

$\begin{matrix} \frac{dh_{v,3,0}(t)}{dt}=\tau_{2}h_{v,2,0}-(\lambda_{h_{v,3,0}}+\mu_{3})h_{v,2,0}+\gamma_{h_{v,3,1}}h_{v,3,1}+[(-1)^{v}\zeta_{h_{1,3,0}}h_{1,3,0}+(-1)^{v+1}\eta_{h_{0,3,0}}h_{0,3,0}]_{t\geq T_{v}} \\ \frac{dh_{v,3,k}(t)}{dt}=\tau_{2}h_{v,2,k}+\lambda_{h_{v,3,\mathrm{k}-1}}h_{v,3,\mathrm{k}-1}-(\lambda_{h_{v,3,k}}+\mu_{3}+\gamma_{h_{v,2,k}})h_{v,3,k}+\gamma_{h_{v,3,k+1}}h_{v,3,k+1}+[(-1)^{v}\zeta_{h_{1,3,k}}h_{1,3,k}+(-1)^{v+1}\eta_{h_{0,3,k}}h_{0,3,k}]_{t\geq T_{v}} \\ \frac{dh_{v,3,\mathrm{K}}(t)}{dt}=\tau_{2}h_{v,2,\mathrm{K}}+\lambda_{h_{v,3,\mathrm{K}-1}}h_{v,3,\mathrm{K}-1}-(\mu_{3}+\gamma_{h_{v,2,\mathrm{K}}})h_{v,3,\mathrm{K}}+[(-1)^{v}\zeta_{h_{1,3,K}}h_{1,3,\mathrm{K}}+(-1)^{v+1}\eta_{h_{0,3,K}}h_{0,3,\mathrm{K}}]_{t\geq T_{v}} \end{matrix}$----(4)

Where $\delta_{vv'}$ is the Kronecker delta function (=0 if $v\neq v'$; =1 otherwise), $f_{v}(t)$ is the fraction of newborns that is vaccinated at time $t$ (=$f_{v}$ if $t\geq T_{vn}\equiv$the time when newborn vaccination starts; =0 otherwise) and the blocks indicated by $t\geq T_{v}$ are only present for times greater than or equal the time when mass vaccination starts. The parameters $\tau_{j}$ and $\mu_{j}$ represent the age-specific maturation and mortality rates, whereas the time dependent quantities $\lambda_{h_{v,j,k}}(t)$ and $\gamma_{h_{v,j,k}}(t)$ are respectively the force of infection from snails and the mortality rate of worm parasites for human hosts in the state $h_{v,j,k}$. The total number of strata $K$is set by requiring $K$>$\max(\lambda)/\min(\gamma)$ so that enough strata are present to reflect the worm distribution in the population at large.

The vaccination rates $\eta_{h_{0,j,k}}$ are implemented such that starting from a selected time ($T_{v}$) and through a selected duration of scale up ($\Delta T_{s}$) a vaccine coverage $C_{h_{0,j,k}}$of the target group $h_{0,j,k}$is reached by the exponentially distributed rates:

$\eta_{h_{0,j,k}}=-\frac{\ln(1-C_{h_{0,j,k}})}{\Delta T_{s}}$ ------------- (5)

The waning of the vaccine is implemented as movement from the vaccinated to the unvaccinated class using the rates $\zeta_{h_{1,j,k}}$.

The above human host system of equations are coupled to a snail infection model comprised of three state variables $x,y,z$ representing, respectively, the fraction of snails that are uninfected, the fraction that are exposed but not shedding (miracidia-exposed), and the fraction that are infected and shedding infectious *Schistosoma* cercaria (patent). In the snail infection model, we assume that the total density of snails in the nearby waters $N_{s}$ is stable thus the different snail fractions add to one:

$x+y+z=1$ ------------------(6)

Also we assume that infected snails in the state $z$ die before recovery and are, in effect, immediately replaced by new uninfected snails (see S1 Fig for cartoon depiction):

$\begin{matrix} \frac{dy}{dt}=\Lambda(t)(1-y-z)-\sigma y \\ \frac{dz}{dt}=\sigma y-\psi z \end{matrix}$ ---------(7)

where $\Lambda(t)$ represents the total force of infection per snail from human hosts, and the parameters $\sigma,\psi$ represent, respectively, the rates at which exposed snails become patent and at which patent snail die.

Human-snail systems are coupled by their respective forces of infection $\lambda_{h_{v,j,k}}(t)$ and $\Lambda(t)$. The forces of infection per human hosts $h_{v,j,k}$ is proportional to the number of patent snails in nearby waters and are given by:

$\lambda_{h_{v,j,k}}=\alpha_{h_{v,j,\mathrm{k}}}z(t)(1-SE\delta_{v1})$ ---------(8)

where $\alpha_{h_{v,j,k}}$is the underlying transmission rate from snails to humans at which, hypothetically, fully 100% infected snails in nearby waters would successfully establish one adult worm parasite in any single human host in the $h_{v,j,k}$strata. These rates are composite parameters and comprise many transmission related parameters such as the rate of exposure to unit surface area of fresh water, snail density in water surface and the probability to establish an adult parasite per unit exposure per host $h_{v,j,k}$. In the SWB framework $\lambda_{h_{v,j,k}}(t)$represent the rate at which human host $h_{v,j,k}$ moves to higher adult parasite burden $h_{v,j,k+1}$, increasing the burden by $\Delta\omega$ adult parasites. The vaccine efficacy of reducing susceptibility to parasite accumulation is given by $SE$.

The snail force of infection is proportional to the total number of eggs released by all human hosts at a given moment in time and is expressed by the following equations:

$$\Lambda(t)=\sum_{v,j} \Lambda_{v,j}(t)$$

-------------(9)

where $[x]$ indicates the nearest integer of $x$ (the floor of $x$). The underlying transmission rate from humans to snails is given by $\beta_{h_{v,j,k}}$and it is the rate at which one egg released per unit time from the population in stratum $h_{v,j,k}$would successfully establish infection in the fraction of snails that is susceptible. Like $\alpha_{h_{v,j,k}}$these rates are also composite parameters that encompass the rate of exposure to (or contamination of) water by human hosts $h_{v,j,k}$ , snail density and the probability of establishing a snail infection per unit exposure per snail. The quantities represent the mean number of mated female parasites and the mean number of eggs produced per mated female parasite (fecundity) by the human host $h_{v,j,k}$. Thus, gives the mean number of eggs produced by the human host at a given time. While this is a time independent quantity for a given host $h_{v,j,k}$, the number of hosts $h_{v,j,k}$is a dynamic variable that varies with time. It is important to note that the assumed number of mated parasite females in the first worm stratum $h_{v,j,0}$ amounts to, which indicates that, in our model, some individuals could be infected but not producing any eggs (these are in stratum $k=0$ and have parasite burden $<\Delta\omega$).

The vaccine efficacy of reducing parasite fecundity is given by the parameter $FE$. Thus far, we have described the parametrization of the vaccine efficacies $SE$ and $FE$. The vaccine efficacy of increasing worm mortality $ME$ enters in the model via the parameters $\gamma_{h_{v,2,k+1}}$. These parameters are given by:

$\gamma_{h_{v,2,k}}=k\gamma_{v,j}=k(1+ME)\gamma_{0,j}$ ---------(10)

We assume that the life span of worm parasites ($\frac{1}{\gamma_{0,j}}$) does not depend on age and is about 5 years [4]. Because we account for vaccination status in the equations above and we assume that the worm accumulation does not depend directly on the existing host parasite burden the quantity $\alpha_{h_{v,j,k}}=\alpha_{0,j}$ only depends on age. Similarly, by assuming that $\beta_{h_{v,j,k}}$is proportional to $\alpha_{h_{v,j,k}}$because they both depend on the contact rates of human hosts with nearby waters then:

$\beta_{h_{v,j,k}}=\beta_{0}\alpha_{0,j}$ --------------(11)

This leaves us with the following uncertain parameters that need to be specified by calibrating the model against egg release data:

$\rho_{0,j}$, $k_{0,j}$, $\alpha_{0,j}$, $\beta_{0}$

The $\alpha_{0,j}$ parameters vary by age according to the contact rates with water. For the coastal Kenyan community modeled here, we used median values from previously published calibration of the basic model without vaccination [5]. To obtain diverse endemic levels we fix the relative contact pattern across age groups and consider variation in *Schistosoma* acquisition and transmission across different communities by varying the contact pattern using a multiplication factor.

# Combined treatment and vaccine model

To capture the effect of administering mass treatment in combination with vaccination, we assumed that treatment was administered to the infected population in combination with, and at the same frequency as that of vaccination. Thus, while we track vaccinated and unvaccinated individuals, the entire infected population at the time of vaccination experiences a relative increase in the worm parasite mortality amounting to treatment efficacy of praziquantel, increasing worm mortality $TE_{m}$ over a period of 28 days.

# The rate of worm accumulation in the model

The population rate of acquiring new worms (worms per person per year) for the age-stratified community is given by the quantity:

$\hat{\lambda}=\Delta\omega\sum_{v,\mathrm{j}} \sum_{k=0}^{k=K-1} \lambda_{h_{v,j,k}}h_{v,j,k}/\sum_{v,j,k} h_{v,j,k}$ ----------------(12)

# The number of people needing to be vaccinated to avert an additional one worm infectious burden

The number of persons who need to be vaccinated in order to avert the accumulation of one worm ($\Xi$) after $t$ years of vaccination is given by the equation:

$\Xi=\frac{V(t)}{W_{0}(t)-\mathrm{W}_{V}(t)}$ -------------------(13)

Where:

The number of individuals vaccinated up to time $t$ $\equiv V(t)$=$\int_{T_{v}}^{t} \sum_{j,k} \eta_{h_{0,j,k}}(t)h_{0,j,k}(t)dt$

The number of new worms accumulated up to time $t$ $\equiv W(t)$ =$\int_{0}^{t} \Delta\omega\sum_{v,j} \sum_{k=0}^{k=K-1} \lambda_{h_{v,j,k}}(t)h_{v,j,k}(t)dt$

and $W_{V}(t)$ and $W_{0}(t)$ represent the number of new worms accumulated up to time $t$ , with and without (baseline) vaccination.

# Model parameterization

Table S2 Text lists model inputs and their sources.

In the model, the population was stratified into compartments according to vaccination status (vaccinated or unvaccinated), age (children 0-4 years old, school-aged children 5-14 years old, young adults 15-24 years old, and older adults 25+ years old), and worm burden with dynamical distribution over strata following a stratified worm burden (SWB) approach (see [2,3,5]).

In the SWB framework, human hosts are divided into worm burden strata $\{h_{k}:k=0,1,...\}$ defined by increments of worm step $\Delta\omega$ with population in $h_{k}(t)$ carrying adult worms in the range $k\Delta\omega$ to $(k+1)\Delta\omega$ at time $t$. Transitions between adjacent strata represent the processes of worm accumulation and death. We ignored the short time interval between worm acquisition and the later adult stage when worms are able to mate. The mating worms produce eggs that are shed into the environment by human hosts, which contaminates nearby waters by infecting fresh water intermediate host snails. The rate of worm accumulation and worm fecundity (number of eggs per mated worm per sample) were assumed to depend on age of host. Worm fecundity was also assumed to decrease with increasing parasite burden (see Table S2 Text).

Demographic data on age-specific mortalities were from Kenya’s demographic and health surveys (KDHS) [6–8], the baseline *in vivo* mortality rate of worm parasites was 0.2 years (reciprocal of 5 years lifespan [4]) and the maximum fecundities of worms (ignoring crowding effect for parasites) were assumed to decrease with host age and were 45, 32, and 11 for years of human host age: <14, between 15 and 24, and ≥25; respectively. For all human host ages, the maximum fecundity was assumed to drop exponentially with the number of parasitic worms due to a crowding effect with a threshold parasitic worm number of 120 worms [5].

We used a simple Susceptible-Exposed-Infectious (SEI) compartmental model for snails with three compartments representing snail’s status of infection: susceptible, pre-patent, and patent. The fraction of patent snails at endemic levels was set in the baseline scenario to ~1% as reported for coastal Kenya in a study that conducted regular snail sampling [9] by adjusting a parameter controlling the movement from pre-patent to patent stages. With the implicit assumption that patent snails die to be instantly replaced by susceptible snails we fixed the snail density with time.

# References

1. Gurarie D, King CH, Wang X. A new approach to modelling schistosomiasis transmission based on stratified worm burden. Parasitology. 2010;137: 1951–1965. doi:10.1017/S0031182010000867

2. Gurarie D, King CH. Population biology of Schistosoma mating, aggregation, and transmission breakpoints: more reliable model analysis for the end-game in communities at risk. PloS One. 2014;9: e115875.

3. Gurarie D, King CH, Wang X. A new approach to modelling schistosomiasis transmission based on stratified worm burden. Parasitology. 2010;137: 1951–1965. doi:10.1017/S0031182010000867

4. Anderson RM, May RM. Herd immunity to helminth infection and implications for parasite control. Publ Online 06 June 1985 Doi101038315493a0. 1985;315: 493–496. doi:10.1038/315493a0

5. Gurarie D, Yoon N, Li E, Ndeffo-Mbah M, Durham D, Phillips AE, et al. Modelling control of Schistosoma haematobium infection: predictions of the long-term impact of mass drug administration in Africa. Parasit Vectors. 2015;8: 1–14.

6. Government of Kenya. National Council for Population and Development (NCPD), Central Bureau of Statistics (CBS) (Office of the Vice President and Ministry of Planning and National Development [Kenya]), and Macro International Inc (MI). 1999. Kenya Demographic and Health Survey (KDHS) 1998. Calverton, Maryland; 1998.

7. Government of Kenya. Central Bureau of Statistics (CBS), Kenya Ministry of Health (MOH), and ORC Macro. 2004. Kenya Demographic and Health Survey (KDHS) 2003. Calverton, Maryland; 2003.

8. Government of Kenya. National Bureau of Statistics (KNBS) and ICF Macro 2010. Kenya Demographic and Health Survey (KDHS) 2008-09. Calverton, Maryland; 2009.

9. Sturrock RF, Kinyanjui H, Thíongo FW, Tosha S, Ouma JH, King CH, et al. Chemotherapy-based control of schistosomiasis haematobia. 3. Snail studies monitoring the effect of chemotherapy on transmission in the Msambweni area, Kenya. Trans R Soc Trop Med Hyg. 1990;84: 257–261. doi:10.1016/0035-9203(90)90278-M
