# Supplementary material for: Quantitative assessment of the impact of partially protective anti-schistosomiasis vaccines
Source: PLoS Negl Trop Dis. 2017 Apr 14;11(4):e0005544. doi: 10.1371/journal.pntd.0005544 (PMC5406007; doi:10.1371/journal.pntd.0005544)
Supplement: S2 Text — (DOCX) [file pntd.0005544.s002.docx]

**Table S1: Model inputs**

| Parameter | Value | Source/comments |
| --- | --- | --- |
| Age-specific mortalities (per person per year) | | |
| Children (age 0-4) | 0.074 | [1] |
| School-aged (age 5-14) | 0.0067 | [1–3] |
| Young adults (age 15-24) | 0.003 | [1–3] |
| Older adults (age ≥25) | 0.03 | [1–3] |
| Population density ($H_{0}$) | 1000 | Representative number of residents in a village. Other values in the range (500-10000) were studied in sensitivity analyses |
| Patent snail initial prevalence (seed) | 1% | Choice was based on [4] |
| Snail's rate of patency parameter (/year) ($\sigma$) | 0.06 | Rate associated with ~1% of snails being patent. Other values in the range (0.03-0.12) were studied in sensitivity analyses. |
| Patent snails turnover rate (/year)  ($\psi$) | 5.3 | Corresponding to a median survival of 9 weeks. Other values in the range (0.5-5 months) were studied in sensitivity analyses |
| The number of worms accumulated per contact between a human and patent snails ($\Delta\omega$) | 10 regardless of human host age | Assumed. Other values in the range (5-15) were studied in sensitivity analyses |
| Threshold parasite number above which crowding effect cause fecundity to drop | 120 worms regardless of human host age | [5] |
| Worm mortality rate in untreated or vaccinated human hosts (per parasite per year) | 0.2 regardless of human host age | Corresponds to an estimated life span of 5 years[6] |
| Worm mortality rate in human hosts treated with PZQ (per parasite per year) | 18.1 regardless of human host age | Corresponds to a surviving fraction of 25% of worms 4 weeks after PZQ administration [7]. This corresponds to an efficacy in killing worms equal to 8936% |
| Maximum fecundity per mated worm parasite inside human host of age $j$[[1]](#footnote-1) (eggs/mated worm parasite/sample) | | |
| Children and school-aged ($\rho_{0,0}$, and$\rho_{0,1}$) | 45.0 | From median values found in [5] |
| Young adults ($\rho_{0,2}$) | 32.0 | From median values found in [5] |
| Older adults ($\rho_{0,3}$) | 11.0 | From median values found in [5]. Other values in the range (1-45) were studied in sensitivity analyses |
| Underlying transmission coefficients from snail to man of age $j$^*^ | | |
| Children ($\alpha_{0,0}$) | 10 | From median values found in [5] |
| School-aged ($\alpha_{0,1}$) | 30 | From median values found in [5] |
| Young adults ($\alpha_{0,2}$) | 30 | From median values found in [5] |
| Older adults ($\alpha_{0,3}$) | 10 | From median values found in [5] |
| The relative transmission rate from humans to snails ($\beta_{0}$) | $5\times10^{-8}$ | Estimated. This parameter was varied between (50%-400%) of this default value in sensitivity analyses |

# References

1. Government of Kenya. National Bureau of Statistics (KNBS) and ICF Macro 2010. Kenya Demographic and Health Survey (KDHS) 2008-09. Calverton, Maryland; 2009.

2. Government of Kenya. National Council for Population and Development (NCPD), Central Bureau of Statistics (CBS) (Office of the Vice President and Ministry of Planning and National Development [Kenya]), and Macro International Inc (MI). 1999. Kenya Demographic and Health Survey (KDHS) 1998. Calverton, Maryland; 1998.

3. Government of Kenya. Central Bureau of Statistics (CBS), Kenya Ministry of Health (MOH), and ORC Macro. 2004. Kenya Demographic and Health Survey (KDHS) 2003. Calverton, Maryland; 2003.

4. Sturrock RF, Kinyanjui H, Thíongo FW, Tosha S, Ouma JH, King CH, et al. Chemotherapy-based control of schistosomiasis haematobia. 3. Snail studies monitoring the effect of chemotherapy on transmission in the Msambweni area, Kenya. Trans R Soc Trop Med Hyg. 1990;84: 257–261. doi:10.1016/0035-9203(90)90278-M

5. Gurarie D, Yoon N, Li E, Ndeffo-Mbah M, Durham D, Phillips AE, et al. Modelling control of Schistosoma haematobium infection: predictions of the long-term impact of mass drug administration in Africa. Parasit Vectors. 2015;8: 1–14.

6. Anderson RM, May RM. Herd immunity to helminth infection and implications for parasite control. Publ Online 06 June 1985 Doi101038315493a0. 1985;315: 493–496. doi:10.1038/315493a0

7. Cioli D, Pica-Mattoccia L, Basso A, Guidi A. Schistosomiasis control: praziquantel forever? Mol Biochem Parasitol. 2014;195: 23–29. doi:10.1016/j.molbiopara.2014.06.002

1. The symbol $j$is used here to denote the four age groups:$j$=0 for children (0-4 years old), $j$=1 for school-aged (5-14 years old), $j$=2 for young adults (15-24 years old) and $j$=3 for older adults (≥25 years old). [↑](#footnote-ref-1)
