# Supplementary figures and images for: Quantitative assessment of the impact of partially protective anti-schistosomiasis vaccines
Source: PLoS Negl Trop Dis. 2017 Apr 14;11(4):e0005544. doi: 10.1371/journal.pntd.0005544 (PMC5406007; doi:10.1371/journal.pntd.0005544)

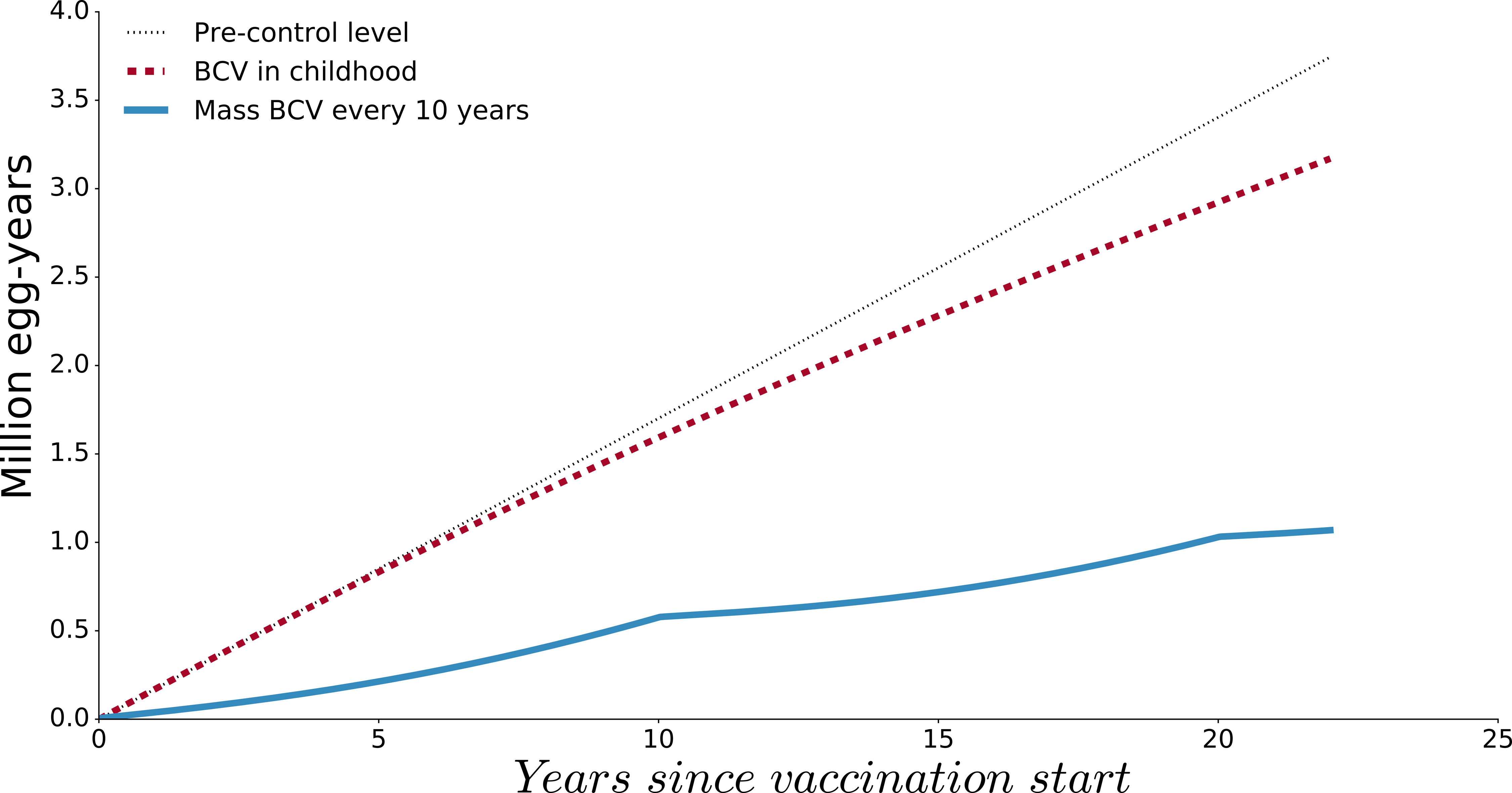

Supplement: S2 Fig — The vaccine’s efficacies are SE = FE = ME = 80% and it confers a mean duration of protection (D) of 10 years. Two schedules are shown: mass vaccination every 10 years for three rounds of vaccination (“Mass BCV every 10 years”) and universal vaccination of newborns (“BCV in childhood”). The assumed size of the population is 1000. (TIF) [file pntd.0005544.s004.tif]

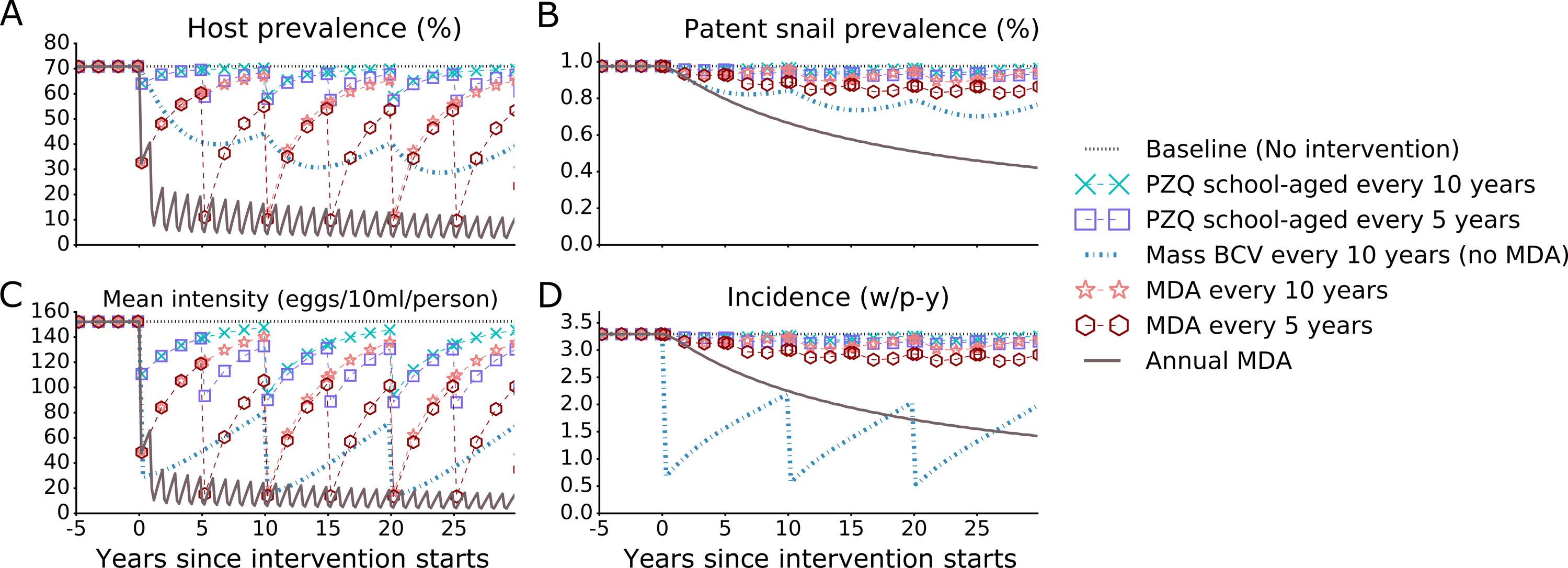

Supplement: S3 Fig — The panels indicate the impact of vaccination on (A) human host prevalence, (B) patent snail prevalence, (C) mean intensity of human infection (eggs/10-ml sample/person or e/s/p) and (D) incidence measured as the number of new worms acquired per person-year (w/p-y). For comparison we include a scenario of base case vaccine (BCV, a vaccine with 80% efficacy and 10-year durability offered in mass campaigns every 10 years) without MDA. This is shown by a blue dotted line. (TIF) [file pntd.0005544.s005.tif]

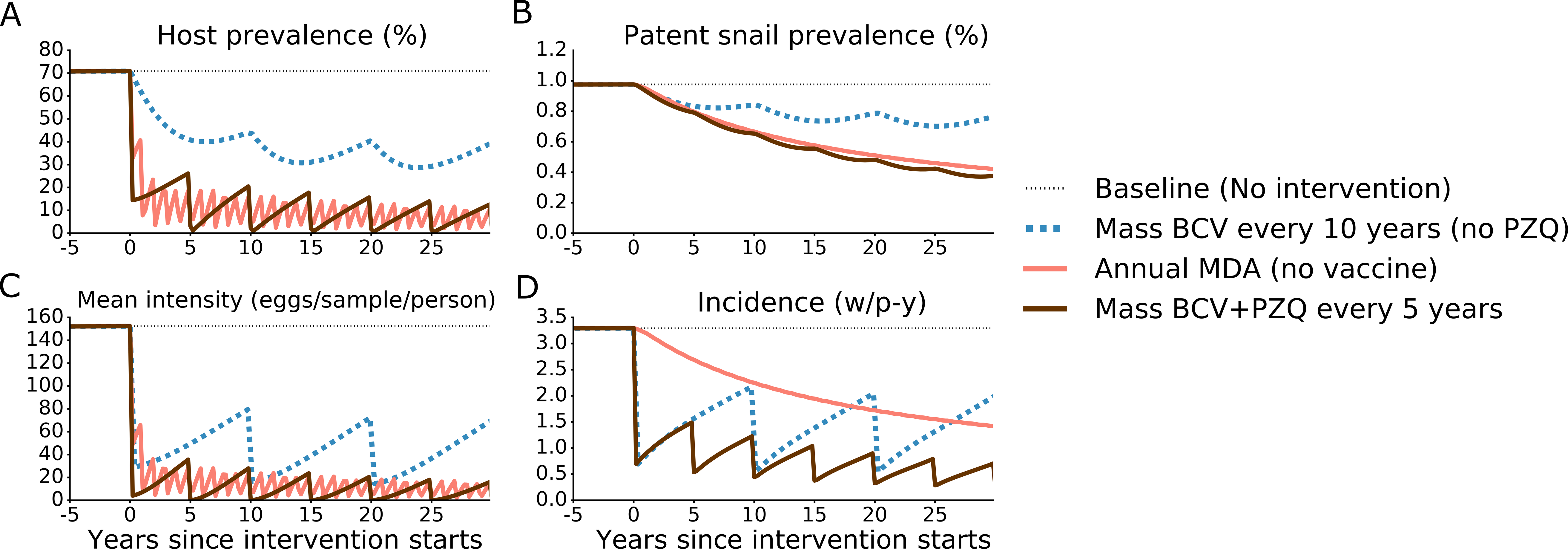

Supplement: S4 Fig — The panels indicate the impact of different interventions on (A) human host prevalence, (B) patent snail prevalence, (C) mean intensity of human infection (eggs/10-ml sample/person or e/s/p) and (D) incidence measured as the number of new worms acquired per person-year (w/p-y). For comparison we include a scenario of base case vaccine without PZQ as a blue dotted line (BCV, a vaccine with 80% efficacy and 10-year durability offered in mass campaigns every 10 years). (TIF) [file pntd.0005544.s006.tif]

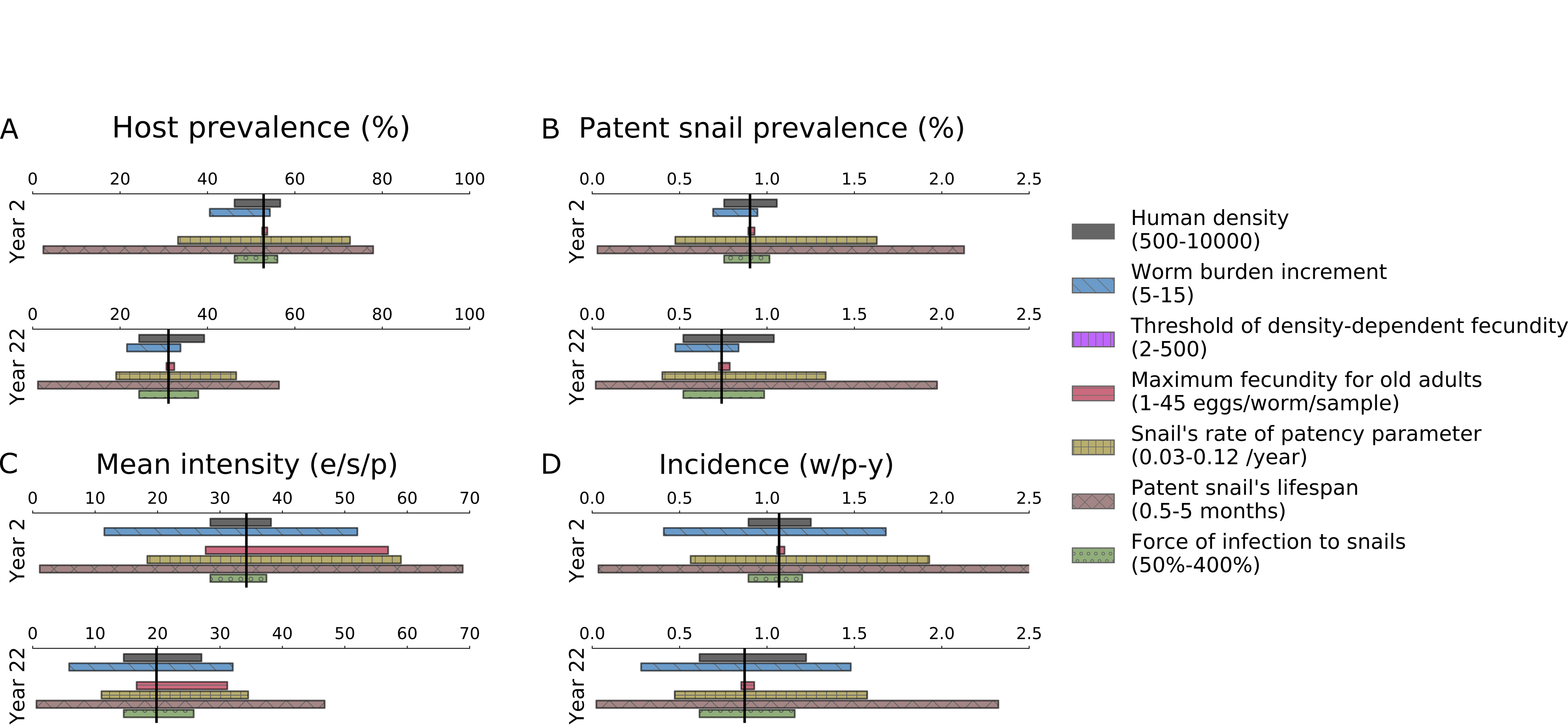

Supplement: S5 Fig — The panels indicate model prediction sensitivity in (A) human host prevalence, (B) patent snail prevalence, (C) mean intensity of human infection (eggs/10-ml sample/person or e/s/p) and (D) incidence measured as the number of new worms acquired per person-year (w/p-y). (TIF) [file pntd.0005544.s007.tif]
